# OGS2: genome re-annotation of the jewel wasp *Nasonia vitripennis*

**DOI:** 10.1186/s12864-016-2886-9

**Published:** 2016-08-25

**Authors:** Alfredo Rago, Donald G. Gilbert, Jeong-Hyeon Choi, Timothy B. Sackton, Xu Wang, Yogeshwar D. Kelkar, John H. Werren, John K. Colbourne

**Affiliations:** 1 Environmental Genomics Group, School of Biosciences, University of Birmingham, Birmingham, UK; 2 Department of Biology, Indiana University, Bloomington, IN USA; 3 Cancer Center, Department of Biostatistics and Epidemiology, Medical College of Georgia, Georgia Regents University, Augusta, USA; 4 Department of Organismic and Evolutionary Biology, and FAS Informatics Group, Harvard University, Cambridge, USA; 5 Department of Molecular Biology and Genetics, Cornell Center for Comparative and Population Genomics, Cornell University, Ithaca, USA; 6 Department of Biostatistics and Computational Biology, University of Rochester Medical School, Rochester, USA; 7 Department of Biology, University of Rochester, Rochester, USA

**Keywords:** Genome annotation, Hymenoptera, Parasitoid wasp, Transcriptome, Alternative gene splicing, Gene duplication, Histones, Protein evolution

## Abstract

**Background:**

*Nasonia vitripennis* is an emerging insect model system with haplodiploid genetics. It holds a key position within the insect phylogeny for comparative, evolutionary and behavioral genetic studies. The draft genomes for *N. vitripennis* and two sibling species were published in 2010, yet a considerable amount of transcriptiome data have since been produced thereby enabling improvements to the original (OGS1.2) annotated gene set. We describe and apply the EvidentialGene method used to produce an updated gene set (OGS2). We also carry out comparative analyses showcasing the usefulness of the revised annotated gene set.

**Results:**

The revised annotation (OGS2) now consists of 24,388 genes with supporting evidence, compared to 18,850 for OGS1.2. Improvements include the nearly complete annotation of untranslated regions (UTR) for 97 % of the genes compared to 28 % of genes for OGS1.2. The fraction of RNA-Seq validated introns also grow from 85 to 98 % in this latest gene set. The EST and RNA-Seq expression data provide support for several non-protein coding loci and 7712 alternative transcripts for 4146 genes. Notably, we report 180 alternative transcripts for the gene *lola*.

*Nasonia* now has among the most complete insect gene set; only 27 conserved single copy orthologs in arthropods are missing from OGS2. Its genome also contains 2.1-fold more duplicated genes and 1.4-fold more single copy genes than the *Drosophila melanogaster* genome. The *Nasonia* gene count is larger than those of other sequenced hymenopteran species, owing both to improvements in the genome annotation and to unique genes in the wasp lineage.

We identify 1008 genes and 171 gene families that deviate significantly from other hymenopterans in their rates of protein evolution and duplication history, respectively. We also provide an analysis of alternative splicing that reveals that genes with no annotated isoforms are characterized by shorter transcripts, fewer introns, faster protein evolution and higher probabilities of duplication than genes having alternative transcripts.

**Conclusions:**

Genome-wide expression data greatly improves the annotation of the *N. vitripennis* genome, by increasing the gene count, reducing the number of missing genes and providing more comprehensive data on splicing and gene structure. The improved gene set identifies lineage-specific genomic features tied to *Nasonia*’s biology, as well as numerous novel genes.

OGS2 and its associated search tools are available at http://arthropods.eugenes.org/EvidentialGene/nasonia/, www.hymenopteragenome.org/nasonia/ and waspAtlas: www.tinyURL.com/waspAtlas.

The EvidentialGene pipeline is available at https://sourceforge.net/projects/evidentialgene/.

**Electronic supplementary material:**

The online version of this article (doi:10.1186/s12864-016-2886-9) contains supplementary material, which is available to authorized users.

## Background

The jewel wasp *Nasonia vitripennis* belongs to the superfamily Chalcidoidea, which is a vast group of hymenopterans that consists mostly of parasitoids that deposit their eggs in or on other arthropods. Parasitoids play an important role at controlling insect populations and are used extensively as an alternative to pesticides [1]. *Nasonia* is the genetic model system for parasitoids and a model for evolutionary and developmental genetic studies [2, 3]. As an hymenopteran, it provides a study system with naturally occurring haploid stages (males) and is a non-social relative to the ant and bee lineages, having diverged from them approximately 170–180 MYA [4, 5]. The *Nasonia* genus includes at least four species [6] that are partially to completely reproductively isolated by the bacterial parasite *Wolbachia,* yet can be crossed after its removal [7, 8], allowing the study of speciation from both a genetic [9–12] and non-genetic [13] perspective. The draft genome assembly of *N. vitripennis* was published in 2010 [4]. At that time, it provided a first comparative study of hymenopteran genomes with reference to the honeybee, *Apis mellifera*. The *N. vitripennis* genome project also included genome sequences for the cross-fertile species *N. giraulti* and *N. longicornis*, which were aligned to the *N. vitripennis* reference genome assembly. Utilizing information from these genomes, advancements have been made in areas as diverse as behavioural ecology [14], speciation [10, 11], immune responses [15] and DNA methylation [16].

In the coming years, projects such as the i5K and 1KITE [5] will continue to deliver new insect genomes and transcriptomes to the research community, with the goal of improving genomic knowledge for this most speciose animal clade [17]. Expanding the taxonomic breadth and number of well annotated genomes is important to develop new research avenues, and several quality measures are necessary for the accurate interpretation of comparative genomic, transcriptomic and epigenomic data [18]. Completeness (the number of reported genes compared to the actual number of genes in the organisms’ gene set) is one such measure; an incomplete gene set may exclude the true causal genes responsible for trait variation in quantitative genetic analyses and confound the interpretation of genome-wide association studies. The accuracy and reliability of gene models are equally important for genetic and genomic studies. Erroneous models can arise either from the fragmentation of true genes or by falsely joining neighboring genes (also termed fused or chimeric models, not to be confounded with their biological counterparts) because of mismatched splice sites, missing exons, or the addition of spurious exons. False models are especially problematic for the functional study of genes by misrepresenting their true expression levels. Finally, an accurate annotation of untranslated regions is required to investigate post-transcriptional regulation. Untranslated regions (UTRs) consist of 5′ and 3′ terminal portions of the mRNAs, as well as introns that are removed from the final mRNA via splicing. UTRs are functionally relevant since they are often targets for regulatory mechanisms such as microRNAs mediated regulation [19, 20], ribosomal binding affinity [21] and transcript localization [22].

The quality of genome annotations is improved by using more sequence data of gene transcripts. These data often expand the initially reported gene repertoires, indicating that (except for a few model species) current gene inventories are still far from completion. The gene numbers and accuracy of annotations for model species have generally increased over decades of work (e.g. 10 % more genes and 200 % more alternates for *Arabidopsis* over 15 years [23]). Species specific, targeted strategies are employed to refine the annotated gene sets. For example, by applying specific targeted solutions to the technical challenges of annotating the honey bee genome (largely because of its unusual base composition), its initial count of ca 10,000 genes [24] increased to a more acceptable gene count of 15,314 [25]. Improving a gene set’s quality however does not necessarily require targeted strategies. Integrating multiple gene-model construction algorithms and incorporating novel expression data can often provide sufficient evidence to improve existing models while also uncovering new loci and their variants. This is especially true if the source data are tissue-specific or include novel environmental conditions and developmental stages, which are likely to reveal the expression of specialized genes or transcripts [26, 27]. For example, the *Anolis carolinensis* gene set was updated in 2013 by adding tissue and embryonic specific RNA-Seq datasets, which provided sufficient new data to increase the overall gene count from 17,792 to 22,962 genes and from 18,939 to 59,373 transcripts – an increase of 29 % and 210 % respectively [28]! These case studies indicate that we are still far from reaching the point of diminishing returns on investments at improving the annotation of eukaryote genomes. As such, the genomics community is aware that updates to integrate novel expression and sequence data must remain a priority in order to provide a more accurate representation of the real biological background of animals.

The construction of a biologically accurate gene set for any species is a complex process, where all data sources of gene evidence should be compared to resolve discrepancies; for each possible artifact there are biologically true equivalents to consider (gene fusions, functional fragments from partial duplication events, exons that become disrupted or functional during evolution). Each data source of evidence can also introduce measurement errors while each gene modeling or assembly method can produce flawed models at a non-deterministic frequency. Therefore, a consensus approach is perhaps the best way at resolving discrepancies among gene structures and to eliminate errors. This approach is implemented by the EvidentialGene method [29] described below.

We report on a more comprehensive official gene set for *N. vitripennis* (OGS2), which vastly improves our understanding of its genome biology. Since its public release in 2012 [30], OGS2 has been used in a number of studies [11, 14–16, 31] and as a resource for comparative genomics (e.g., through databases such as OrthoDB [32, 33]). Here we describe *N. vitripennis* OGS2 in detail and compare it to the earlier annotation set using several quality measures. We use OGS2 for a comparative analysis of gene family expansion and sequence evolution with reference to other hymenopteran genomes. Finally, we reveal the usefulness of the novel gene set by presenting a multi-factorial analysis of the features that characterize alternatively spliced genes, demonstrating that genes with annotated isoforms are characterized by longer transcripts, greater number of introns, slower rate of protein evolution and lower probabilities of duplication when compared to genes with no alternate transcripts.

## Results and discussion

### Source data and gene model construction

RNA-Seq produced 187,823,326 single-end sequence reads and included 124,188 paired-end and 51,665 single-end EST sequences from previously published ([4]; SOM) and unpublished data sets. The reads were mapped onto the draft Nvit_1.0 genome and assembled into gene transcripts using three methods (Cufflinks, Velvet and PASA) with six different sets of parameters producing between 46,259 and 242,217 *de-novo* constructed mRNA (Table 1). Twenty one thousand, six hundred and one (21,601) and 10,426 constructed mRNA aligned to the final gene models by 10 % and 95 % overlap, respectively (Table 1). The multiple-constructed mRNAs for each gene were evaluated by three classes of evidence-based criteria, which were then combined to calculate weighted-evidence scores resulting in a final pick of 44,164 transcripts of which 7,837 are alternate splice variants (Table 1). These *Nasonia* transcript assemblies were also used to construct NCBI’s gene set (NCBI build 2.1; http://www.ncbi.nlm.nih.gov/genome/guide/wasp/release_notes.html).

**Table 1:** Gene evidence sources for *Nasonia vitripennis* OGS2. Mapping results of ESTs and RNA-Seq reads with >95 % coverage of length >100 bp to the assembled *N. vitripennis* genome (Nvit_1.0) using three mapping software and six parameters. An average of 2.5 % of reads are multiply mapped by GSNAP, measured over 8 RNA-Seq libraries. Number of constructed transcript assemblies matching the final gene model by 10 % and 95 % sequence overlap is also indicated

	RNA assemblies	Mapped to genome	10 % of gene	95 % of gene
Cufflink 10	46,259	40,853	12,386	4902
Cufflink 08	71,761	56,640	14,317	5287
Velvet p2	121,672	95,360	16,190	7706
Velvet p3	151,038	116,591	17,556	7851
Velvet p4	242,217	122,194	16,406	6874
PASA	69,805	69,805	13,099	6253
All genes			21,601	10,426
Alt. Transcripts	7,837			
RNA read counts	EST paired	124,188		
	EST single	51,665		
	RNA-Seq single	187,823,326		

During the development of this updated gene set, several advances in the use of complex gene evidence for producing and selecting accurate and complete gene sets were tested and employed. We used an automated method of selecting gene models that best fit the range of gene evidence, including reference proteins, expressed sequence reads (EST, RNA-Seq), and whole genome tiling array expression. Our method also included a per-locus assessment and classification of the agreements among the various types of gene evidence, because each gene modeler produces locus-specific models that best fit the evidence. Testing and refining the evidence scores, with expert assessment and direction, is a core component of this process.

We found that expression evidence from tiling arrays and RNA-Seq accurately track gene structures, by sharply rising at the start of exons and dropping at their ends, *on average* (Additional file 1: Figure S1). Therefore, combining both sources of evidence improve the delineation of gene structures. We learned during our gene modeling efforts that tiling array expression data were problematic when using available modeling tools, despite the high average accuracy for gene structure, as they only consist of exon data, without defining individual gene end points nor intron splice sites at nucleotide resolution. As a result, genes modeled with strong contributions from tiling expression were often aberrant (Additional file 1: Figure S2), with UTRs much longer than coding sequences, overlapping two or more reference protein models, and extending through introns defined using other evidence. While average tiling expression matches gene structure well, for individual loci that exon signal is obscured by lack of precise gene end point and intron signals, which are however available from RNA-Seq reads and assemblies.

RNA reads and assemblies were more reliable for precisely defining gene structures by providing evidence in four forms: *(1)* reads mapped to the genome (exon parts), *(2)* introns from splice-mapped reads on genome, *(3)* full or partial transcripts assembled onto the genome and *(4)* assembled *de-novo* structures without the genome. These all contributed different and important aspects of gene structure evidence for modeling. Intron-exon splice sites are particularly reliable evidence of gene structures; each intron is measured by expressed reads that are splice-mapped to a genome, where the accuracy of the splice point increases with read coverage over that point. On the other hand, assembled transcripts can capture a gene fully, without further modeling; however, they also exhibit more errors of fragmentation or over-extension (gene joins) that must be assessed using other sources of evidence. *De-novo* assembled transcripts have the unique advantage of being unaffected by large breaks in genes on the genome, long introns and transposons, and mis-assemblies. Unlike the gene predictor algorithms, transcript assembly methods are also not focused on modeling coding sequences, and thus better reconstruct non-coding transcripts. The main drawback of the available RNA-Seq data for this study is that they were generated by early-generation instruments and chemistries (Illumina and Roche-454), which produced sequence reads of lower quality and quantity than desired for obtaining many complete gene assemblies. Yet these were usefully combined with other gene evidence and predictor methods. The complete EvidentialGene construction pipeline software, along with the *Nasonia*-specific configurations and methods, is available for public use at http://arthropods.eugenes.org/EvidentialGene/nasonia/ and https://sourceforge.net/projects/evidentialgene/ [30, 34].

A final set of 36,327 distinct loci, selected by EvidentialGene methods was compared to other available and draft *Nasonia* gene sets (Tables 2 and 3). The predicted models include UTRs based on expression data and genome gene signals. Putative long non-coding genes (lncRNA) from the transcript assemblies – those with weak coding potential and no homology to reference proteins – were retained in the full gene set. The models and EST evidence were assessed with PASA for valid alternate transcripts. Gene proteins were annotated with Uniprot descriptions, and classified by evidence scores, including transposable elements.

**Table 2:** Summary of the improved Official Gene Set (OGS2) comparing all gene constructions to good constructions having expression and/or homology evidence and to the previous OGS1.2 gene models. Percentages are of the total number of genes for the set

Summary Statistics	OGS2 All Models	OGS2 Good Models	OGS1.2 Final Models
Genes	36,327	24,388	18,850
Protein coding genes	25,725 (71 %)	24,388	15,566^a^
Non-coding genes	3,997 (11 %)	0	0
Transposon protein genes	6,605 (20 %)	385^a^	2,935^a^
Single transcript genes	32,079 (88 %)	20,243 (83 %)	18,759 (99.5 %)
Genes assigned to ortholog^b^	15,176 (42 %)	15,173 (62 %)	--
Transcripts	44,164	32,101	18,941
Alternative transcripts	7837	7712	91
Mean isoforms per gene	1.22	1.32	1
Complete proteins	41,256 (93 %)	30,521 (95 %)	18,941 (100 %)
Median transcript length	1571 bp	1603 bp	1176 bp
Median CDS length	777 bp	981 bp	1032 bp
Transcripts with UTR	41,313 (94 %)	30,512 (95 %)	5264 (28 %)

^a^2,935 OGS1.2 models are classified with strong homology to transposon proteins during OGS2 work, 385 models with expression and other insect homology but also transposon homology were retained in OGS2 “good” model set

^b^5,763 additional genes of OGS2 have significant protein homology, but are not assigned as orthologs in OrthoMCL orthology analysis, 3,454 of 24,388 “good” models lack significant homology, but have expression evidence

**Table 3:** The types of evidence and levels of support for *Nasonia vitripennis* gene sets (OGS2 and others). Sequence-level statistics for the different types of evidence are given as proportions of the gene sets that are validated. Gene structure level statistics (ESTgene, Progene, RNAgene) are counts of the number of models that reach three structure level agreements. Homology level statistics are counts of the number of models and proportions matching proteins of reference species and paralogous (same species) proteins. See Methods section for details on the evidence types and the statistics that were measured

Evidence	Available evidence	Statistic	OGS1.2	Evidence-prediction set	OGS2	OGS2 Good genes	NCBI RefSeq	Full-length RNA-Seq assembly
EST	18 Mb	Seq. Overlap	0.506	0.814	0.768	0.715	0.672	0.724
Protein	26 Mb	Seq. Overlap	0.674	0.696	0.729	0.693	0.616	0.612
RNA	46 Mb	Seq. Overlap	0.381	0.551	0.599	0.54	0.468	0.571
RefSeq	17 Mb	Seq. Overlap	1	0.934	0.958	0.908	0.857	0.839
Intron	66,593	Splices Hit	0.846	0.965	0.981	0.969	0.903	0.975
TAR	75 Mb	Seq. Overlap	0.292	0.850	0.533	0.443	0.37	0.386
Transposon	28 Mb	Seq. Overlap	0.168	0.282	0.406	0.099	0.009	0.039
ESTgene	10,194	Perfect	2737	3996	4952	4900	3631	4293
ESTgene	10,194	Equal 66 %	3491	5059	6283	6198	4284	5187
ESTgene	10,194	Some	6263	9940	11,313	11,157	7123	8373
Progene	44,040	Perfect	4808	6713	8048	8010	6215	4935
Progene	44,040	Equal 66 %	7759	12,217	14,046	13,837	9003	8567
Progene	44,040	Some	11,563	18,173	21,759	19,718	10,861	18,457
RNAgene	28,016	Perfect	6004	9531	14,899	13,804	8502	28,016
RNAgene	28,016	Equal 66 %	8173	13,552	18,829	17,608	10,202	28,016
RNAgene	28,016	Some	11,933	19,602	24,936	22,179	12,258	28,016
Homolog	11,683	Matches	16,174	16,669	23,994	17,341	11,950	13,187
Homolog	11,683	Found	10,426	10,593	11,683	11,683	9323	9650
Homolog	11,683	Bits/Amino Acid	0.449	0.424	0.416	0.455	0.562	0.558
Paralog		Matches	12,843	14,503	19,423	12,576	7904	10,520
Paralog		Bits/Amino Acid	0.459	0.45	0.564	0.517	0.554	0.635
Genome		Coding Seq.	28 Mb	31 Mb	36 Mb	29 Mb	10 Mb	16 Mb
Genome		Exon Seq.	29 Mb	52 Mb	70 Mb	45 Mb	24 Mb	24 Mb
Genome		Gene count	18,941	23,605	36,327	24,388	12,989	20,926

Finally, 24,388 constructions were chosen to be “good models” (Table 2), having the best match to EST and protein homology evidence. Models excluded from the “good” set include: *(1)* those with expressed RNA assemblies but with weak or no coding potential, *(2)* most of those with significant homology to known transposon proteins, and *(3)* those with minor or no expression and protein evidence from the quality assessment. However, 385 genes having homology to putative transposon proteins but also with expression and homology to other insect species genes were retained as an indeterminate subset annotated as “expressTE”. We used the “good models” set for all downstream analyses, but note instances where the remainders include some genes of biological value.

### Gene model quality assessment

**Fig. 1:**
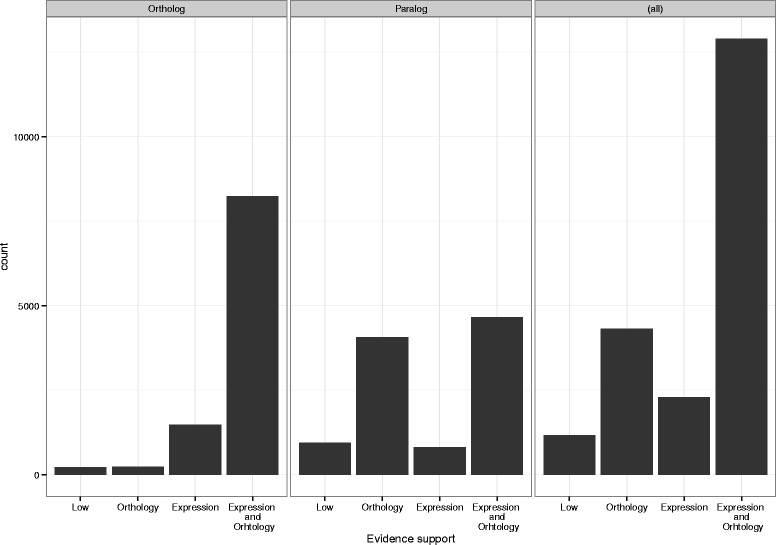
Number of genes with strong (>2/3 overlap) or medium (>1/3 overlap) support from sequence orthology, evidence of transcription, or both. Panels show the source of evidence for genes within the ortholog and paralog subsets and the whole OGS2

We compared the relative contribution of both expression and homology to the construction of gene models in OGS2. Details of this evidence scoring of gene models are described in the Methods section, with results summarized in Table 3 for each evidence type, and is here presented as percentages of evidence that overlaps or is recovered in gene models on the genome assembly. Expression data supports 17,925 genes (74 % of OGS2) at strong (>2/3 overlap) or medium (>1/3 overlap) levels of evidence. Strong or medium homology support is present for 17,238 genes (71 % of OGS2). The intersection of strong and medium support from both lines of evidence contains 12,912 genes (53 % of OGS2, Fig. 1), suggesting a high degree of convergence (*p*-value = 2E-14, Fisher’s exact test).

While still significant (*p*-value = 1E-8, Fisher’s exact test, *N* = 13,861), the level of convergence between expression and orthology support decreases to 44 % for the subset of duplicated genes, likely due to a reduced relative support of expression data (Fig. 1). The decrease in expression support can be explained by a more restricted expression profile for paralogs, which often arises after gene duplication events [35]. Therefore, further transcriptomic data from different tissue types and conditions should increase the level of convergence between the orthology and expression sets. Conversely, genes without duplicates show greater convergence between orthology and expression support (81 % of 24,388 genes, Fig. 1).

Most of the 24,388 OGS2 genes that map to the *N. vitripennis* genome assembly (Additional file 2: Table S1) also map to the genome assemblies of sibling species *N. longicornus* and *N. giraulti* [4] using GMAP [36]; 664 do not map to *N. longicornus*, and 735 do not map to *N. giraulti* (391 are missing in both, yet 50 of these have non-wasp orthologs). All 4,141 high identity paralog loci from *N. vitripennis* map to assemblies of both siblings, though some are overlapping loci (Additional file 2: Table S1). The majority of paralog mapping patterns are the same for all 3 species (i.e., their relative positions are shared for all three species): 83 % (3442/4141) of the paralogs for all species, 99 % (4098/4141) of the paralogs for 2 or 3 species. The differences include both real biological differences and assembly errors. Of the 2481 paralogs on separate scaffolds of the *N. vitripennis* genome, 328 overlap first paralog spans in other species, therefore may be missing or mis-assembled. Of 239 tandemly arrayed paralogs in *N. vitripennis*, 128 are also tandem in other species, 101 are on separate scaffolds in other species, and 69 overlap first paralog spans in other species (ie. missing or mis-assembled).

We also report that 3558 genes (15 % of OGS2) have no homology support and are therefore annotated only by means of expression data, and that 1818 genes (7.5 % of OGS2) have no expression support and are therefore annotated only by means of orthology matching. Eight hundred and thirty-three (833) genes in OGS2 are expert-curated including 38 that span different scaffolds, odorant genes, and other cases that could not be annotated automatically. Finally, 374 transcripts have complete proteins from transcript assemblies that do not match genome sequence due to genome gaps and frame-shifts.

### Gene set completeness

We assessed the level of completeness of the OGS2 gene set using OrthoMCL to classify genes into orthologous gene families that are common to arthropods (Tables 4 and 5). The comparison of genes among nine species indicates that OGS2 is equally or more complete than the other insect gene sets, having fewer missing gene families, and similar numbers of orthologous gene groups and single copy orthologs. Additionally, OGS2 reveals that *Nasonia* has twice the number of duplicated genes than *Drosophila melanogaster* or *Tribolium castaneum*, both with homology (in-paralogs) and without (unique duplicates), plus a greater number of unique singletons. Measures of protein sizes and alignment score (Table 5) indicate that OGS2 genes are larger on average than genes from other versions of the *Nasonia* annotated gene sets, yet near to the *Apis mellifera* ortholog gene sizes.

**Table 4:** Number of insect genes classified to gene families (GF) that are common among the arthropods by OrthoMCL (ARP9, version arp11u11). Five out of nine insect species are summarized. Dupl and Singl designate the proportion duplicated and singleton genes relative to the median found among insects (Dupl:5000, Singl:10000)

	Gene Families (GF)			Gene Counts					Proportions	
Gene Sets	GF	Ortholog GF	GF missing genes	Genes	Species specific genes	Species specific paralogs	Single ortholog genes	Duplicated ortholog genes	Dupl	Singl
*Nasonia* OGS2	10,293	8983	92	24,296	5446	6686	8239	3925	2.1	1.4
*Apis*	8591	8560	170	10,145	987	88	8182	888	0.2	0.9
*Harpegnathos*	9633	9291	107	15,029	2943	1567	8710	1809	0.7	1.2
*Tribolium*	8893	8388	116	16,985	4586	2163	7608	2628	1.0	1.2
*Drosophila*	8464	7636	187	14,289	2824	2556	6994	1915	0.9	1.0

**Table 5:** Gene set quality measurements, including deviation of protein size from the group median, and maximal bit score per species in pairwise comparisons within the arthropod orthology groups. The bit score measures both gene model artefacts of alternative gene sets within species, and evolutionary divergence. Protein sizes may be more evolutionarily conserved, and may detect artefacts across and within species^a^

Gene set	Average homology bitscore	Protein size deviation from median	Percent shorter than 2 standard deviations from median
*Nasonia* OGS2	727.6	−7.7	3.2
*Nasonia* NCBI	722.3	−7.8	2.7
*Nasonia* OGS1.2	683.5	−12.7	4
*Apis*	733.9	−0.3	2.4
*Harpegnathos*	694.3	−30	7.3
*Tribolium*	552	−26.1	4.5
*Drosophila*	508.7	54.5	1.3

^a^For each orthology group, the median protein size of all genes among the species within the group is determined. Then for each species gene set, the maximal BLASTp bit score of a gene within that group is recorded as metric #1, and the protein size difference from the group median of that maximal match is recorded as metric #2. These metrics are averaged for all groups per species, and reported as average bit score, as average size deviation, and as percentage of size outliers (2 standard deviations below median sizes). These gene set quality measurements are provided by the Evigene scripts: “eval_orthogroup_genesets.pl” and “orthomcl_tabulate.pl”. Partial gene models are a common artefact of draft gene sets, indicated by both a negative deviation from group median sizes, and larger percentage of outliers. A similar calculation is part of the OrthoDB methodology [108]

The transcript assemblies contain 62 orthologous gene groups that are not included within OGS2 because these transcripts are only poorly positioned onto the *Nasonia* genome assembly. These may be included in a more complete gene set as transcript assemblies, but are not yet part of this genome-mapped OGS2 gene set (Additional file 2: Table S2). A total of 75 orthologous gene groups are missing in *Nasonia* but present in 9 other insect genomes (Additional file 2: Table S3).

We also used the OrthoDB method to independently assess completeness. We counted the number of missing conserved single-copy genes that are otherwise present among the sequenced Arthropoda (Benchmarking Sets of Universal Single-Copy Orthologs [BUSCO] in OrthoDB Release-6), as well as the multi-copy *Nasonia* genes that are otherwise classified as single copy in other Arthropoda. For the majority of gene families, there were no discrepancies between the results obtained from OrthoDB and OrthoMCL. Although the BUSCO results suggest that OGS2 lacks 67 of the 3377 (2 %, listed in Additional file 3) conserved ortholog groups, further analyses found all but 27. Conserved families missing in *Nasonia* OGS2 according to OrthoDB can be attributed to *(i)* genome artifacts (10 missing genes were found split across assembly scaffolds, or lost in gaps but found in transcript assembly), *(ii)* gene model artifacts (9 loci were apparent join errors appended to a second gene protein*), (iii)* OrthoDB discrepancies at classifying proteins to families (25 loci were assigned to different gene families by OrthoMCL and by OrthoDB family). Twenty-seven conserved single copy genes are either truly missing or sufficiently diverged to avoid detection. This number is comparable to those in other Arthropoda, which lack a number of BUSCO genes ranging from 3 (*Drosophila erecta*) to 708 (*Strigamia maritima*), with a median of 42.

Experimental evidence supports the lineage-specific gene loss for the three BUSCO genes involved in developmental regulation: *short gastrulation* (*sog*, OG EOG6S4MX5), *spaetzle 3* (OG EOG61C5BT) and *daughters against dpp* (*Dad* or *smad6*, OG EOG69CNQ7). Despite their ultra-conserved status across currently sequenced arthropods, detailed investigations of *Nasonia* development suggest that those genes are truly absent from its genome due to modifications in the BMP signaling pathway [37] rather than because of omissions in the current annotation.

Since genes in the BUSCO set are defined as single-copy in 90 % of 30 arthropod species, we compared the number of duplicated BUSCO genes in OGS2 to estimate the fraction of potential false gene duplications. We counted 141 (4 %) multiple-copy OGS2 of the total 3377 BUSCO single-copy gene families (Additional file 4). Of those, 62 (44 %) are reported as duplicates uniquely for *Nasonia*, 61 for *Nasonia* plus one additional species, and 18 for *Nasonia* plus two other species. Other species have similar rates of duplicated single-copy genes: 78 for *Apis mellifera* and *Harpegnathos saltator*, 96 for *Pogonomyrmex barbatus*, 119 for *Atta cephalotes* (all Hymenoptera), 107 for *Anopheles,* and 437 for *Aedes* mosquitos. *Nasonia* OGS2 is therefore well within the observed range of duplications of BUSCO genes.

To further assess whether the reported duplicates are likely to be false models, we removed the best supported gene from each orthologous group and measured the expression support of the remaining models. One hundred and fifty-three (153) out of 175 genes (87 %) show medium or strong support for expression and only 2 have no expression support. Lineage-specific duplications are supported by the observation that the majority of genes belonging to ultra-conserved ortholog groups display moderate to strong expression, even after removing the most supported duplicate and map to different genomic locations (data not shown).

### Improvements in genome annotation

OGS2 improves our knowledge of the *Nasonia* genome in several ways (Table 2). First, the number of annotated genes climbs from 18,850 to 24,388 (an increase of 29 %). This greater completeness of the *Nasonia* gene set is corroborated by the sharp decrease in Arthropod ortholog groups missing from the *Nasonia* genome. OGS1.2 lacked 609 ortholog groups that are present in all other Arthropoda (OrthoDB Release-5). Only 331 conserved OGs are now missing from OGS2 when compared to the same subset of species (OrthoDB Release-6) and 253 when considering all currently available arthropod species.

The spans of coding exons are very similar between OGS2 and OGS1.2 for 10,583 loci, which have a median percent equivalence of 92 % between both sets. Changes in coding sequences are mostly attributable to error correction such as splitting and merging of models: 1617 original gene models (10 % of OGS1.2) have been split into separate genes in OGS2, while 3555 OGS2 genes (15 % of OGS2) contain a portion of an OGS1.2 split gene, and 494 OGS2 genes result from the joining of two or more OGS1.2 fragment genes (30 from three or more). Moreover, the proportion of genes with UTR extensions is now near complete: 23,069 (95 %) of OGS2 gene models have annotated UTRs compared to only 5,264 genes (28 %) within OGS1.2. These gene models match 98 % of 66,593 intron locations on the genome assembly, identified by multiple reads of expressed RNA (>3; Table 3), compared to 85 % within OGS1.2 and 90 % within NCBI-11 RefSeq. Intron splice sites are strong indicators of genes, including species-specific genes. This measure therefore indicates a high level of gene set completeness, independent of protein homology. Finally, OGS2 dramatically increased the number of annotated transcripts from 91 alternate transcripts in 91 genes (0.5 % of OGS1.2, Additional file 2: Table S4 in [4]) to 7712 transcripts among 4146 genes (17 % of OGS2). Therefore, OGS2 increases the completeness of the reported *Nasonia* gene repertoire and the quality of gene models as well as allowing a first overview of *Nasonia* transcriptional diversity.

The current release also increases the diversity of annotated wasp genes. Of all OGS2 gene models, 12,296 (50 %) could not be assigned a putative function *via* orthology with other annotated genes. Four thousand, six hundred and fifty-six (4656) genes from this subset (38 %) could be assigned to 2334 arthropod orthologous groups, 490 of which (21 %) are present as multiple copy in *Nasonia*. The remaining 7640 genes with no known function are found exclusively in OGS2 and could not be assigned to orthologous groups shared with other arthropods (OrthoDB, release 6). This subset is likely to include both incorrect models and innovations along the wasp lineage. Three thousand, nine hundred and eighty-three (3983) of those *Nasonia*-only genes (52 %) are present as duplicates in OGS2, a proportion that is significantly greater than that reported for the whole genome (fisher’s exact test, *p*-value < 2.2E-16). Of the 7640 lineage-specific genes with no annotated function, 4498 (59 %) have been newly annotated in OGS2.

### Mapping of OGS2 to *Nasonia vitripennis 2.1* genome reference assembly (Nvit_2.1)

To facilitate the broad use of the new OGS2 *Nasonia* gene set, we mapped it to the latest assembly (Nvit_2.1), using the UCSC LiftOver tools. The gene set is almost unchanged when transferred to the newer coordinate system. Out of 226,902 exons in the Nvit_1.0 gene set, 226,441 (99.8 %) can be successfully mapped to the Nvit_2.1 assembly. Focusing on transcript models, we find that 98.7 % of transcript models are identical between coordinate systems (43,590 out of 44,164). Of the 574 transcript models that differed between coordinate systems, 167 have all exons present but with small changes in the length of either exons or introns. For example, one exon is 170 bp shorter in the newer assembly for locus Nasvi2EG031848t1. An additional 155 genes are missing all their exons, and 252 are missing at least one exon but are present as partial models in Nvit_2.1.

In addition to the General Feature Format file (GFF) with gene models in the Nvit_1.0 coordinate system, we also provide a reduced GFF (only exon and CDS features) with features mapped to Nvit_2.1 coordinates, a table with the status of each transcript in the new assembly, and UCSC-style liftOver chains to convert between Nvit_2.1 and Nvit_1.0 (Additional file 5). A relational file matching gene models between OGS1.2, OGS2.0 and NCBI-101 based on genome assembly locations is also included (Additional file 6).

### NCBI 2014 gene annotation of *Nasonia*

When OGS2 was produced in 2011, its quality metrics ranked above *Nasonia* gene sets of NCBI and OGS1.2 (Tables 2 and 3). Since then, the NCBI gene set has improved along with enhancements to NCBI’s Eukaryote Genome Annotation Pipeline [38], producing *Nasonia vitripennis* Annotation Release 101 in 2014 (which we abbreviate as NCBI-101). These improvements partly resulted from greater use of RNA expressed sequences, and improvements at identifying related insect gene sets for consensus orthology. Among this project’s contributions were its RNA assemblies for *Nasonia* that NCBI used for gene modelling.

The NCBI-101 *Nasonia* gene set includes 13,141 protein-coding gene loci, 24,626 transcripts, and 945 noncoding or pseudogenic genes. We compared protein-coding exon spans of the OGS2 genes that were lifted onto assembly Nvit_2.1 with those of NCBI-101 mRNA loci, using exon locations on the newer assembly. Model equivalences are measured as percentage of base overlap of coding-exon and full exon locations on the same genome assembly. These model equivalences are tabulated in Additional file 6. Of the NCBI loci, 12,319 (93 %) genes have at least some equivalence to OGS2 loci; a majority of 8400 (64 %) genes have nearly identical coding spans at > = 95 % equivalence, and 10,820 (82 %) genes are mostly the same (> = 66 % equal). The non-equivalent loci, with no exon overlap, include 11,535 (47 %) of the OGS2 “good” set and 867 (7 %) of the NCBI-101 set, plus 574 OGS2 loci noted above that are not properly located on the Nvit_2.1 assembly.

Protein homology to other insects is very similar for NCBI-101 and the OGS2 gene sets. Of the conserved eukaryotic protein domains in NCBI’s Conserved Domain Database, we find 9165 domains in NCBI-101 and 9347 in OGS2 from 9505 total aligned domains using RPSBlast, having similar alignment lengths (average 233 aa for NCBI-101, 235 aa for OGS2). Among the complete proteins of related species and gene families identified with OrthoMCL (see Methods section), NCBI-101 contains 68 % of the gene families compared to 67 % for OGS2, both with average 85 % alignment to these proteins.

Of the 11,535 non-equivalent OGS2 loci, 85 % are expressed genes with homolog alignments ranging from none to full; the remainder is supported only by protein homology. Expressed paralogs are the most common (6296/11,535, 55 %) subclass. Of 867 non-equivalent NCBI-101 loci, 512 have uncharacterized proteins, and 21 have model exceptions on this genome assembly (frameshifts, mis-maps). Of 339 NCBI-101 loci with characterized products, many are those we identified in the *Nasonia* transcript assemblies that were not located in our genome gene models (Additional file 2: Table S2). Also, 389 of the extra NCBI-101 loci are found within our OGS2 full (“not-good”) gene set; 76 of those are characterized proteins. Recent experiments have demonstrated that these “extra” loci in OGS2 are biologically significant. For example, of the 248 OGS2 genes that are immune responsive [15], 94 (38 %) are not among the NCBI-101 loci. *Nasonia* genes expressed in brain and nervous tissue [31] include 39 of 304 (13 %) not among the NCBI-101 gene set.

### Expanded gene families

Our examination of the updated gene families of OGS2 identified 411 Arthropoda ortholog groups that have duplicated exclusively in the *Nasonia* lineage (4 % of all ortholog groups within OGS2). These groups consist of 1230 genes, of which 599 loci (49 %) have no assigned homolog (Additional file 7). The most frequent category among annotated expanded genes within the “good models” set is that of transposon associated proteins (102 genes, 30 ortholog groups), followed by kinases/phospatases (38 genes, 16 ortholog groups) and odorant receptors (23 genes, 7 ortholog groups). The enzyme 5-hydroxyprostaglandin dehydrogenase (6 paralogs, 2 ortholog groups) also shows an evolutionarily interesting lineage-specific expansion. This protein is essential for male pheromone processing, and is a prime candidate for driving mate selection and speciation, based on positional cloning of genes involved in pheromone differences between *Nasonia* species [11].

### Protein evolution in Hymenoptera

**Fig. 2:**
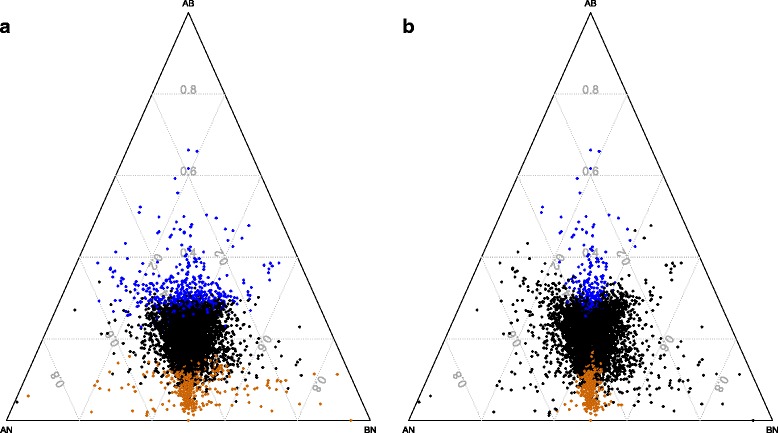
Protein divergence of OGS2 genes against orthologs in other Hymenoptera. Every point represents a gene mapped on three coordinates originating from the corners. Each gene’s distance from a corner is proportional to the average amino-acid distance of orthologs between the two clades. AB = ant to bee distance; AN = ant to *Nasonia* distance; BN = bee to *Nasonia* distance. Diverging genes are highlighted in orange (fast) and blue (slow) as detected by the compound ratio (A) and intersection of ratios (B). See materials and methods for full description

We calculated the sequence divergence of each *Nasonia* gene from its orthologs in both ants and bees. We then selected *Nasonia* genes that have a significantly higher or lower proportion of sequence divergence to ant and bee orthologs when compared to the rest of the *Nasonia* gene set (see Methods section for details). This method identified 504 genes (the most extreme 5 % of the frequency distribution) for both the rapidly and the slowly evolving gene categories (Fig. 2a; Additional file 8).

We also adopted a more stringent approach by measuring the divergence scores of *Nasonia* genes against genes of the ant and bee lineages separately, then selecting only those genes that scored as rapidly or slowly diverging in both. This intersection method identified 596 and 394 genes that have differentially accelerated or slowed evolutionary rates in the *Nasonia* clade, respectively (Fig. 2b; Additional file 8). We note that both methods are unrooted, which therefore identify genes with greater divergence in *Nasonia* relative to bees and to ants, not to the common ancestor of these three lineages.

In all subsets, the most significantly enriched Gene Ontology terms are “nuclear location” for the cellular component category, “DNA/chromatin binding” for the molecular function category and “transcriptional regulation” for the biological process category. These data are consistent with the view that evolution of unique metazoan traits occurs more by changes in transcriptional regulators rather than in structural proteins [39, 40].

### Histone genes

Although histone genes are generally highly conserved, we identified several members of the histone complex with sequences that evolved relatively rapidly in the *Nasonia* lineage. Specifically, we observe a greater rate of sequence divergence for the histone proteins H2A when compared to ant and bee variants. Histone H2A proteins package DNA into chromatin and are implicated in epigenetically mediated gene expression regulation in vertebrates [41–43]. Regulatory variants of H2A histones are also present in the *Apis mellifera* genome [44]. There are currently twenty-four (24) H2A genes within OGS2, 22 of which are assigned to a single ortholog group (OG) (Arthropoda OG EOG6VT4F0) and 18 of which are assigned to a single Hymenoptera group (OG EOG65QGR3). Compared to other Hymenoptera, this ortholog group is more rapidly evolving in *Nasonia* and has a greater number of paralogs: four times greater than *Linepithema humile* (the 2nd highest number with only five copies). However, we cannot rule out that the number of H2A genes in other hymenopterans is underestimated, especially considering the comparable number of H2A genes that are found in other arthropods (e.g. 21 in *Daphnia pulex,* 22 in the *Culex quinquefasciatus*, 22 in *Drosophila melanogaster*). As of now, only two *Nasonia* H2A genes have strong homology with genes within Hymenoptera, while most others have higher scoring sequence similarity matches (using Blast) among vertebrate histones. This pattern can be explained by a lineage specific increase in protein sequence evolution, which would decrease the similarity between histones of *Nasonia* and of other hymenopterans, and therefore increase their relative similarity to those of more distantly related species by a phenomenon called long-branch attraction. Thus, even though the match to vertebrate seems better than to hymenoptera, this result is most likely an artifact, yet is still indicative of a faster evolutionary rate of *Nasonia* histones compared to those of other hymenoptera.

Histone H3 is known to exhibit a wide range of modifications, many of which have known effects on the transcriptional status of the underlying genes [27, 45]. Several *Nasonia* H3 proteins (Hymenoptera OG EOG6R4ZDK) appear to significantly evolve less rapidly when compared to ant and bee orthologs. We find that this apparently slower evolutionary rate of this orthologous group is due to a mis-identification of this OG, which is comprised of at least two different paralogs at the base of the hymenopteran lineage (Additional file 9). One of these putative sub-groups is retained in two copies across all Hymenoptera. The other sub-group is present in 2–4 copies in most Hymenoptera; yet *Nasonia* has 14 copies. The combination of an artefactual fusion of two OGs and unequal representation of *Nasonia* duplicates between the two groups is therefore the cause for an apparent slower relative evolutionary rate; the the correct interpretation consists of a lineage-specific expansion. *Nasonia* also retains an H3 gene of the OrthoDB group EOG62V6ZW, which is shared with other arthropods but not with other Hymenoptera, and and H3 gene of the OrthoDB group EOG6ZCRM6, which is seemingly lost in the bee lineage.

The *Nasonia* H2B histone proteins are encoded by 21 genes; only four are assigned to an ortholog group containing other hymenopteran genes (EOG6Z8X7C of OrthoDB, whereas 8 are assigned to an OrthoMCL group). All genes are diverging at comparable rates while Nasonia’s copy number within this orthology group is similar to that of other hymenopterans (5 in *Pogonomyrmex barbatus* and *Atta cephalotes*). The remaining seventeen H2B histones could not be analyzed by our method, as they are not assigned to other hymenopteran H2B histone gene families (OrthoDB, release 6). Those genes may be mis-identified by the annotation pipelines, yet the NCBI-101 gene set independently annotates 18 of these 21 loci as H2B histone proteins, suggesting that this annotation is supported by available evidence, and may comprise a *Nasonia*-specific expanded histone gene cluster(s). By contrast, the *Nasonia* H1 histone is present as a single copy in the genome with no significant difference in its divergence rate from those of other Hymenoptera.

We found that families of histone modification enzymes have specifically expanded in the *Nasonia* genome: 4 of 38 histone-related gene families (10 %) meet our criteria for lineage-specific expansion (see Methods section). By comparison, expansions are found in only 0.013 % of gene families for the rest of the genome. Our data therefore suggests that the *Nasonia* genome is enriched for histone modification enzymes due of lineage-specific gene expansions (Additional file 2: Table S4; *p*-value = 0.024, Fisher’s Exact test). The finding suggests that histone modification, rather than DNA methylation, may play an important role in the lineage-specific features of epigenetic modulation in *Nasonia*, consistent with findings that DNA methylation does not differ between the sexes in *Nasonia*, nor correlate with epigenetic changes in gene expression [31].

### Non-coding RNA

An early observation from the RNA-Seq and tiling array data sets is an abundance of expression in non-protein coding regions. These poorly annotated regions (in *Nasonia* and in other genomes of well-studied model organisms) require attention, as they are either UTRs of annotated protein coding genes, or putative long non-coding RNA (lncRNA). Our full gene set contains 3,997 putative lncRNA that were recovered from the *Nasonia* transcript assemblies (listed in “OGS2 All models”, Table 2). Among the OGS2 good coding models, 5,450 genes have annotated UTRs that sum to >50 % of their transcript length. The remaining ~40 % of expressed RNA remains to be annotated (Table 3, RNA evidence). Because our genome annotation methods focused on coding regions, resulting in an acceptable number of expected orthologs compared to the proteomes of other species, the remaining expression is likely non-coding. This large fraction of expressed RNA that has yet to be annotated is expected; these are found to exceed protein-coding genes in mammals [46], and to have significant similarities to characterized lncRNAs and UTRs [47].

Long expression spans near conserved coding genes are also observed in the *Drosophila* and *Mus* genomes, including nervous system specific expression, modeled both as long UTRs [48] and as lncRNA [49, 50]. We provide six examples of such long expression spans near *Nasonia* genes along with their presumed orthologs (*ELAV-2 RNA-binding protein*, *calmodulin CaMKI*, *casein kinase II beta*, *odd-skipped*, *dunce/cAMP-specific 3′,5′-cyclic phosphodiesterase*, and homeobox gene *extradenticle*) in *Pogonomyrmex*, *Apis*, *Drosophila* and *Mus* (Additional file 10). These expression spans are annotated as UTRs, sense and antisense lncRNA, or often without annotation. Difficulty at modeling these spans is not unique for *Nasonia*; a benchmark comparison of annotation methods (including those we used) for reconstructing Human and *Drosophila* non-coding genes found that all methods lacked accuracy [51].

Knowledge of these non-coding regions is nevertheless valuable for biological study, even when imperfect. For example, a recent study of *Nasonia* genes expressed in brain and nervous tissue [52] identified 306 OGS2 genes as differentially transcribed for learning in wasps – including *dunce*, *CaMKI* and *ELAV-2* – with their associated long non-coding spans. Among the 3,997 putative lncRNA listed in “OGS2 All models”, 15 are discovered to be differentially expressed for learning [52] (Additional file 10) suggesting a significant role for non-coding RNAs in regulating neuronal development and function [53, 54]. Finally, 322 expressed non-coding regions located upstream of *Nasonia* coding genes are identified across insect genomes [55]. Functional genomic studies will help elucidate the importance of this significant portion of non-coding expression.

### Alternate transcript diversity including *lola* expansion

**Fig. 3:**
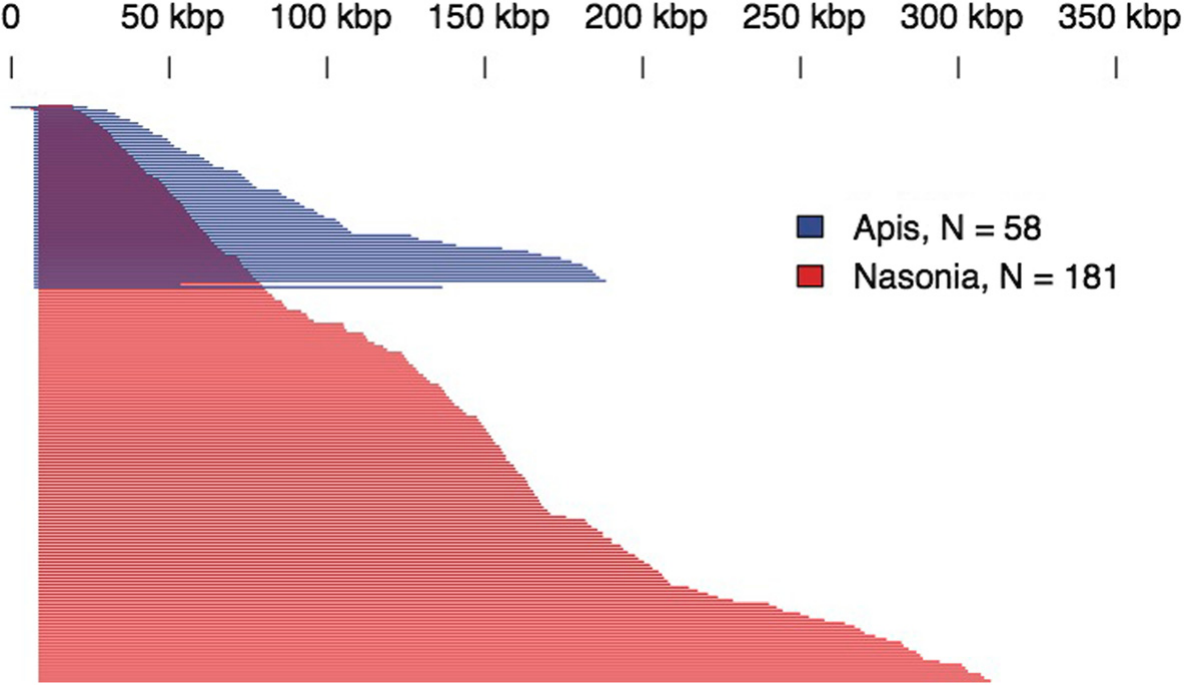
Alternate spliced, expressed introns for gene *longitudinalis lacking* (*lola*) in *Apis* (blue) and *Nasonia* (red). Graph shows intron spans from a common hub exon, in bases on their genomes. The observed 181 introns in *Nasonia* cover 325 kilobases (kbp), and up to 200 kbp in the 58 observed introns in *Apis*. These are regularly spaced 1400 bases apart, related by divergent 3′ exons (one or two) of 500 to 900 bp, which produce different coding sequences and protein isoforms. The tiny blue and red bars at top of figure are short introns that join pairs of 3′ end exons in *lola* gene span. Introns are displayed in size order (y axis), but for a plotting mistake at *Apis* long end

OGS2 includes alternate transcripts assembled from available expressed sequence using genome-mapped assembly and *de-novo* assembly methods. A total of 7712 alternate forms are identified for 4145 genes (17 % of the total reported genes). One thousand, seven hundred and twenty-five (1725) genes (42 %) have at least 3 isoforms, 219 genes (5 %) have at least 6 isoforms and 26 genes have at least 10 isoforms. One gene (*longitudinals lacking* or *lola*) has a notable expansion of over 180 alternate forms, of which 89 are included in the OGS2 gene set. The remaining alternative transcripts are identified by read splice introns. Named for its observable wing phenotype in *Drosophila*, *lola* is also expressed in many tissues and developmental stages, and has a putative role in neuronal development [56]. *Lola* alternate transcripts all share a common 5′ set of six exons, with one hub exon that branches to alternate 3′ coding sequences of 500–900 bp, spanning 350 kb of the genome, with a new alternate each 1400 bases (median). *Apis mellifera* shares this *lola* alternate expansion, with 58 annotated alternates branching over 200 kb from the single hub exon, as shown in Fig. 3. In both species, additional alternates may be discovered with further expression evidence, as the regular spacing in *Nasonia* suggests up to 250 may fit into this region of the genome. Examination of non-hymenopteran insects shows no similarly large expansion for *lola*.

The *Nasonia* gene with the second largest number of isoforms is the neuronal developmental transcription factor *fruitless*, with 17 alternative isoforms. *Fruitless* was already characterized as having an unique gene structure in *Nasonia* compared to dipterans, and its differential splicing is involved in both development and sexual differentiation [57]. Two other *fruitless* paralogs are also reported within OGS2, while no other insect genome shows paralogs for this gene. Other genes with a high number of reported isoforms include mostly transcription factors and various kinases/phosphatases (Additional file 11).

### Evolution of alternative splicing

**Fig. 4:**
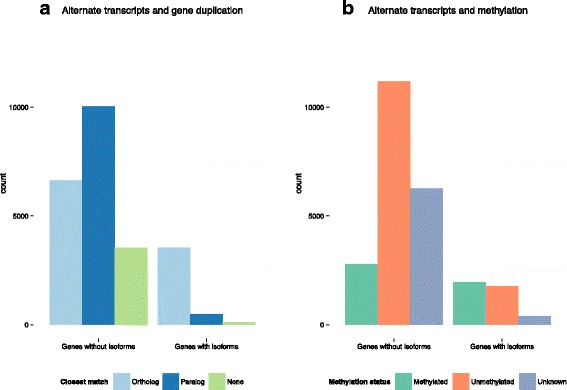
Number of genes with alternative isoforms in OGS2 (**a**) split by presence of paralogs and (**b**) split by methylation in adult females

The augmented number or genes with reported isoforms in OGS2 allowed an examination of factors that contribute to the evolution of this regulatory mechanism. From a total of 4146 genes with reported isoforms, only 476 (11 % of all genes with isoforms, 2 % of OGS2) have annotated paralogs (Fig. 4a). This proportion is significantly less (*p*-value <2.2xE-16, Fisher’s Exact Test) than the product of proportions of genes with alternative transcripts and that of genes with duplicates (17 % × 43 % = 7.3 %). In addition, genes without paralogs also have a greater number of introns than those with duplicate copies in the genome (Kruskal-Wallis rank sum test, *p*-value <2.2E-16 for both strict and broad sense paralogs). Possible interpretations of these patterns are considered in the discussion section below.

Methylation has been proposed as a molecular mechanism for the regulation of alternative splicing in humans [58]. In Hymenoptera, studies of both bees and ants consistently locate methylation target sites at the intron-exon junctions [44, 59, 60]. However, a study on the *Nasonia* methylome [16] reports alternative transcripts in non-methylated genes and no correlation between presence of alternate splicing and methylation status. We re-tested for the overrepresentation of alternative splicing with OGS2 sets of known methylated and known non-methylated genes (reported in [16]) (Fig. 4b). Results indicate a significant overrepresentation of isoforms among methylated genes (*p*-value = 2.2e-16, Fisher’s exact test), with alternative transcripts reported for 41 % of methylated genes, while only 14 % of non-methylated genes have transcript isoforms.

**Fig. 5:**
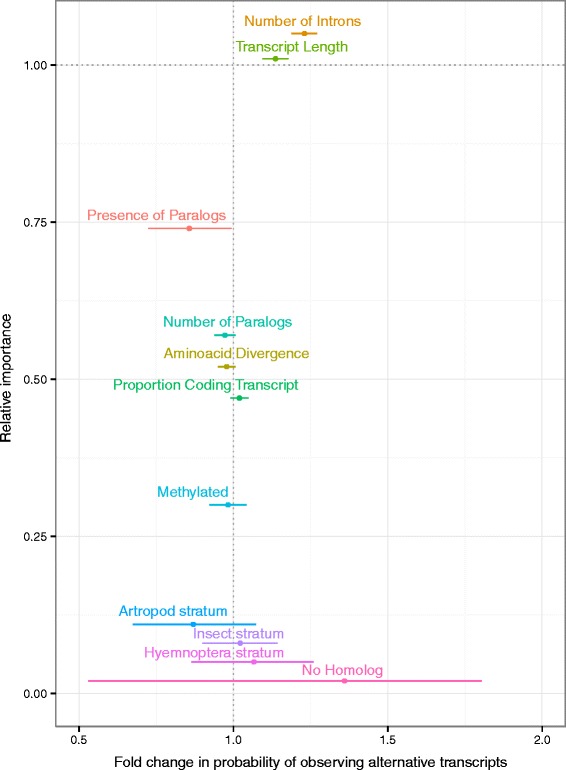
Effect of different factors on the probability of observing alternate isoforms of OGS2 gene models. Factors are ranked by relative importance (y axis). Factors with complete support and levels of the same factor were adjusted for plotting. Effect sizes are shown as the fold change in probability from the intercept (with 95 % confidence intervals). Numeric variables were log transformed prior to analysis

To exclude spurious results due to correlation with unaccounted variables, we fitted a generalized linear mixed model (GLMM) to estimate the probability of observing alternative transcripts in OGS2 genes according to a variety of factors (see Methods section for details). The final statistical model (Fig. 5) is composed of the following co-factors: strict sense paralogy (presence of a reciprocal best match within the genome), number of broad-sense paralogs (OGS2 genes within the same arthropod ortholog group), ratio of *Nasonia*-specific protein evolution within Hymenoptera (see Methods section “Identification of fast- and slow-diverging genes in the *Nasonia* relative to ants and bees”), number of introns, methylation status in adult female and furthest matching ortholog. We also fitted a random error structure to account for individual differences between ortholog groups.

Expression level and intron support are also expected to be main predictors of observed alternative isoforms, since isoforms of genes with greater transcript abundances will be easier to detect via RNA-Seq. We could not include expression and intron support as factors in our analyses due to their high correlation with methylation status (see Methods section, Additional file 12: Figure S5). We therefore restricted our analyses to the subset of genes that have both strong expression and strong intron support (*N* = 5447, Fig. 5).

Results indicate that the number of predicted introns and transcript length are positive predictors of alternative isoforms. Both findings are consistent with recent studies on the *Apis* transcriptome [60]. The presence of introns enables the evolution of alternative splicing, since the latter requires differential inclusion of exons. The role of transcript length is more difficult to interpret. It is possible that genes with longer transcripts simply reflect better annotation quality. Alternatively, longer transcripts may allow for longer intronic sequences, which may facilitate the emergence of alternative splicing by providing a greater number of targets for the generation of novel splice sites or by switching from the intron signaling mechanism to the more error prone exon signaling mechanism [61]. We explicitly included coding sequence to transcript length ratios among factors of interest to study these effects. We found that the proportion of coding transcript sequence (CDS/transcript length) is less well supported than transcript length itself (47 % relative importance versus 100 %). Furthermore, genes with higher proportions of non-coding sequence have a lower probability of displaying alternative transcripts. Even by assuming a role for intronic to exonic sequence length proportions, we find that shorter exons are prevalent among spliced genes, contrary to both the novel splice site and exon definition modes of new isoform generation. We should however note that the prevalence of long introns flanking alternative exons appears to be primarily driven by isoforms that comprise a minor proportion of all splice variants of a gene [61]. It is therefore possible that the slight skew towards genes with low proportions of intronic sequences might be driven by issues in annotating low-abundance isoforms rather than by biological constraints.

Our initial genome-wide analyses detected a correlation between methylation and alternative splicing. However, we observe alternative transcripts for non-methylated genes as well as methylated genes. This finding indicates that methylation is not necessary for alternative splicing in *Nasonia*. Furthermore, after focusing on the subset of genes with strong expression and intron support, methylation status in adult females is only weakly correlated with presence of isoforms (relative importance 30 %).

We find low support for a negative correlation between *Nasonia*-specific sequence divergence and probability of observing alternative splicing. Methylated genes are known to have a slower rate of protein sequence evolution in *Nasonia* [16], while the presence of paralogs often increase protein evolutionary rates by releasing *pleiotropic* constraints on individual gene copies. Yet, rate of sequence evolution and lack of isoforms remained correlated, even after controlling for the effect of methylation and paralogy (relative importance 52 %). This finding suggests that, despite the relatively low level of support, the inverse correlation between protein sequence evolution and alternative splicing may be direct result, rather than being derived from indirect correlations, and is consistent with studies of the *Apis* genome [60].

Both measures of paralogy (by reciprocal best hits or number of genes within the same arthropod ortholog group) retained a moderate level of support (74 % and 57 % respectively) when compared to other factors. Presence and number of paralogs are correlated with a lower probability of observing alternative transcripts. Since we performed all our analyses on the subset of genes with strong expression support, we can dismiss an effect due to the relatively lower expression support available for duplicated genes (see Fig. 1). The relatively large confidence intervals of the estimated effect of this factor on the probability of observing splicing of a given gene may either indicate a weak effect or result from the under-representation of paralogs in our subset (6 % of the “good expression” gene set *versus* 43 % of OGS2).

Finally, we tested whether isoforms are observed more or less frequently amongst genes which emerged at a specific taxonomic level by using furthest phylostratigraphic match as a proxy for gene age [62]. While average probabilities decrease with gene age, this trend was not validated as statistically significant (data not shown). Furthermore, no single gene age category significantly alters the probability of observing alternative splicing in its assigned genes (relative importance: 0.07).

The inverse relationship between alternative splicing and gene duplication in particular is consistent with observations on the evolution of mammalian model species’ genomes [63]. There are currently several competing models that explain the negative correlation between gene family size and number of isoforms.

The “function sharing” model hypothesizes that duplication events reduce the selective pressure to maintain alternative transcripts in both gene copies [64]. This model is based on the assumption that both paralogs and isoforms provide equal opportunities for functional diversification. The reduced selective constraint would lead to the reciprocal loss of isoforms and subfunctionalization of the gene copies [65]. Such a scenario had been proposed for the Dscam genes in Arthropoda [66]. The function-sharing model predicts that genes will gradually accumulate isoforms that are lost shortly after duplication events.

By contrast, Roux and Robinson-Rechavi [64] proposed an “age-dependent” model, in which the inverse correlation between duplication and gain of isoforms is not direct but rather arises independently because of structural properties. Short gene length could be advantageous for whole gene duplication, while genes with an already high number of exons will have a higher propensity towards single exon duplication due to replication and recombination errors [64]. The lower numbers of isoforms for genes with duplicates would thus result from the different rates of accumulation of isoforms and duplicates rather than loss of redundant transcripts. This hypothesis has been criticized in depth [67].

Finally, the underlying equivalence between the diversification potential of duplication and alternative splicing assumed by both the function-sharing and the age-dependent models is refuted by [68]. This finding suggests that a gene’s probability of having isoforms rather than duplicates might be less dependent on its structural properties and more dependent on the different adaptive potential of the novel proteins generated by two diversification modes, or functional constraint. Our analyses support longer transcripts and high numbers of exons as predictors of the presence of isoforms. While this is in agreement with the age-dependent model, we do not find a significant correlation between age of a gene family and the presence of isoforms. This could be either be caused by an actual lack of correlation, inaccurate dating [69] or by the fact that the divergence from the most recent outgroup (~180 MYA) is sufficiently great that every new family gains at least one detectable isoform.

Absence of duplicates has moderate support as a predictor of splicing, even after controlling for the structural properties of genes. Together with the lack of support for gene family age, this observation is congruent with the predictions of the function-sharing model. However, we must point out that a true test to falsify the function-sharing model would require testing the significance of the date from last duplication event, which we could not measure with our dataset. Comparisons between the sibling species *N. giraulti* and *N. longicornis* are especially suited to this task, as they provide a sufficiently short timescale to assess transcriptome changes lead by duplication when compared to more basal Hymenoptera.

Since we lack estimates on the potential functional overlap of duplicates and isoforms in the genes we analyzed, we could not explicitly test the independent model. However, the fact that we observe a strong effect of structural gene properties runs contrary to the expectation of a process driven by their different potential to generate adaptive variants.

In conclusion, while we find no evidence for age itself being a determinant of the presence of isoforms, we do find strong support for structural gene properties. This might be explained by an hybrid model in which the final outcome is determined both by the propensity of a gene to produce either isoforms (or duplicates), and by their differential fixation because of their adaptive potential (independent model) or overlap (function-sharing model).

We must point that our study assesses the presence or absence of isoforms, rather than their number, and only considers the subset of highly expressed genes, which might have different selective pressures than restricted ones. Our choices are necessary to provide a fair comparison, since lowly expressed genes have intrinsically lower probabilities of having observable isoforms and the number of isoforms is likely to increase as more diverse RNA samples are sequenced. However, they also skew our analysis towards a non-random subset of genes, which might be subject to different selective pressures. As such, tackling a truly comprehensive analysis of splicing and duplication in the *Nasonia* genome will require more sequencing efforts.

### Community resources for *Nasonia* genomics

Several information resource projects support the use of *Nasonia* for genomics investigations, reviewed by Lynch [3]. Gene set improvements of OGS2 are available at the Hymenoptera Genome Database (HGD) [70] and more recently at WaspAtlas [71]. The HGD provides genome map views and BLAST sequence searches for *Nasonia*, including this OGS2 gene set, and 8 other Hymenoptera species. WaspAtlas offers gene annotation and functional information searches of *Nasonia* gene sets including OGS2, integrating expression and DNA methylation annotations. This OGS2 gene set along with associated gene evidence and alternate gene sets are also available with genome map views and BLAST sequence homology searches through the EvidentialGene project of euGenes genome database [30, 34]. NCBI provides genome map views, sequence and gene annotation searches [38] for their annotations of *Nasonia*. With a growing wealth of genome information, the value of these resources will improve where they can manage to integrate and sensibly organize such data as RNA sequence expression studies, DNA methylation data, proteomics, new genomic data, and cross-integrate with the improving genomics data of related species.

## Conclusions

OGS2 provides a major quantitative and qualitative update to the toolbox for *Nasonia*’s genomics research. Better-defined UTRs enable the study of post-transcriptional regulation via targeting of small RNAs. Novel reported isoforms provide a more accurate representation of gene expression. We also highlight interesting areas for future molecular biology research using this organism, such as histone modification. Furthermore, we provide an estimate of the most unique traits of the *Nasonia* genome when compared with other Hymenoptera, which can assist the discovery of genetic mechanisms underlying the typical features of this lineage.

The advances in gene annotation for OGS2 are notable today, however as gene evidence accumulates in the future, new and improved gene sets will need to be constructed until a verifiably complete and biologically accurate gene set is produced. Transcriptomic data in the form of high quality and inexpensive RNA-Seq is now the leading form of gene evidence for most genome projects, surpassing gene prediction and mapping of reference gene proteins. Along with abundant high quality RNA-Seq for the model *Drosophila*, *Tribolium*, and other insects, the *Apis mellifera* gene set has recently been improved by addition of several billion paired reads, sufficient for the assembly of all but the weakly expressed genes. This approach has been employed at NCBI for updated genome-based models, and at EvidentialGene with RNA-only assemblies. The RNA assemblies may well surpass genome-modeled genes for orthology completeness as well as species-unique completeness [72].

As a proof of concept, all of the novel data that enabled the annotation improvements made by OGS2 are derived from functional genomics methods (RNA-Seq, tiling arrays and ESTs). Transcriptomic data can thus improve genome annotation, even when the underlying genome assembly is frozen. As shown by the publication of results from the modENCODE *Drosophila* project [73], new genes and transcripts are discovered, even for a genome that has been intensively investigated for over half a century. Our modeling estimated that 50 % of all *Nasonia* loci may possess alternative transcripts, comparable to the 57 % observed from the *Drosophila* transcriptome [26], whereas we recovered alternates from RNA assemblies at only 17 % of all loci. Therefore, even though it is unlikely that the addition of novel data will drastically increase the gene count for the *Nasonia* genome, we expect an increase in the number of reported isoforms with the addition of stage, tissue and condition specific transcriptomes. Perhaps more importantly, new data will increase the quality of gene models, where RNA transcript assemblies will validate and improve gene structures, an unresolved subset of which we believe are fragments or gene joins, and will provide further evidence for intron/exon patterning.

Our phylogenetic analyses were restricted in scope to the portion of the genome that could be assigned to an ortholog group, and its interpretation hindered by the large number of genes of unknown function. In order for the genomics of this organism to be better linked to its biology, there is a pressing need for more functional studies tailored to *Nasonia*’s unique features. Genome wide association studies and quantitative trait loci are especially complimentary for this purpose, as they provide a first connection between the well-defined transcriptionally active regions and biologically relevant traits [74, 75]. As a final note, OGS2 is currently rich in models that have little support. These lowly supported models might prove to be a valuable resource for future studies on the unique features of the wasp lineage, as their current status as low-level support loci could either be indicative of a restricted expression pattern or of a recent evolution or emergence in the hymenopteran phylogeny.

## Methods

We constructed gene models by using software methods that incorporate various sources of biological evidence for genes, including transcriptional data from RNA-Seq and tiling-path microarrays and sequence homology with genes described in other species. We performed model quality assessment to select the best gene model per locus and to compare gene sets, using the same gene evidence plus additional sources. After quality assessment, we performed error and discrepancy analyses followed by updated gene set selection in a negative feedback fashion to minimize errors. All selected gene models are supported by some kind of evidence; *ab-initio* predictions without gene evidence are not included in OGS2. A small set of problem genes were manually curated and corrected by expert examination of evidence.

### Gene evidence from expressed transcripts

Total RNA samples for sequencing were collected from whole embryos, pupae, whole adults, adult heads and adult abdomens using the extraction and purification protocol described in [15]. Single-end sequencing libraries were created using the TruSeq chemistry by Illumina following the manufacturer’s instructions. Sequencing was performed on both the GAIIx and HiSeq 2000 Illumina instruments with single-end read lengths of 40, 51 and 80 base pairs. The sequences were deposited at NCBI as BioProject PRJNA219398. Expressed Sequence Tags (EST) from four normalized cDNA libraries – which contributed to the OGS1.2 annotation – were also used in gene construction (accession numbers GE352825-GE467204 and ES613911-ES651267). The library construction and sequencing procedures are described in the Supporting Online Material for [4].

RNA from short and longer reads were assembled into long mRNA transcripts using both genome-mapped assembly (PASA, Cufflinks) [76, 77] and *de-novo* assembly (Velvet/Oases) [78, 79] (Table 1). *De-novo* assembly combined paired-end EST with short read RNA-Seq, whereas PASA only assembled ESTs and Cufflinks only assembled short RNA-Seq reads because of software limitations. We used Cufflinks v1.0.3 and v0.8 with default options, PASA v2.2011 with standard options and Velvet v1.1.05, oases v0.1.22 with options -ins_length_long = 400 -conserveLong yes -min_pair 2, and kmer values 27 and 31. EST and RNA-Seq were mapped onto the draft genome sequence with GSNAP [80] for assembly by PASA and Cufflinks. The *de-novo* assembled transcripts were mapped onto the draft genome sequence with GMAP [36], and incorporated into further gene construction as transcript evidence. Longest open reading frame (ORF) proteins were computed from *de-novo* transcripts, and used in gene orthology assessment and genome assembly discrepancy analyses. Intron evidence was collected from properly spliced reads and transcripts mapped onto the genome assembly; the number of spliced reads per intron location was used as a quality score. We found a total of 66,595 intron locations supported by 3 or more reads, including 1100 introns longer than 20 kbp (285 kbp maximum) supported by at least 10 reads.

### Gene evidence from expression tiling array

We used whole genome tiling-path microarrays with tile spacing of 20 bp to discover transcribed *Nasonia* loci. We extracted total RNA from samples of 5 different life stages, 0–10 h embryos, 18–30 h embryos, 51–57 h larvae, 1-day yellow pupae (little to no red eye pigment), and 1 day post-eclosion adults. We used six replicates per sample, averaging 400 individuals per replicate for embryos, 300 for larvae, 20 for pupae and 20 for adults. Samples were extracted in Trizol (Invitrogen, cat #15596-026) then processed and expression data produced at the Indiana University Center for Genomics and Bioinformatics using previously published methods [81].

Tiling array expression analyses result in exon-like spans, called transcriptionally active regions (TARs), from runs of adjacent expressed 50 bp tiles (Table 6). The log-normalized intensity of replicated tile array signals is primary expression evidence for TARs. Both genome tiling and RNA-Seq expression track gene exon structures well (Additional file 1: Figure S1) suggesting their suitability for gene modelling. TARs were used as exon-like evidence in gene predictions in two ways: as input to AUGUSTUS predictor in the form of exon hints (genome span scores) and as input to exonerate cDNA mapping to gene structures, in combination with other evidence (Table 3, Additional file 1: Figure S2).

**Table 6:** Genome tiling array expression gene evidence. TAR = Transcriptionally Active Regions representing runs of adjacently expressed 50 bp isothermal probes on a genome-wide tiling path microarray [4]

Expression group	TAR exons	Unique TARs	Exonerate gene models
Adult female	1,139,061	29,626	46,402
Adult male	1,165,881	20,625	49,344
Embryo 10 h old female	700,773	21,704	33,286
Embryo 10 h old male	677,712	6788	31,408
Embryo 18 h old female	781,163	13,268	31,342
Embryo 18 h old male	813,130	15,662	33,612
Larva female	670,292	7173	29,442
Larva male	667,030	3814	28,284
Pupa female	1,246,557	16,563	51,858
Pupa male	1,322,223	15,769	54,119
Ovaries	631,449	7113	27,483
Testes	658,960	21,449	30,348

### Gene evidence from related species proteins

Gene homology evidence for the gene construction pipeline was collected from 220,000 proteins of 2 ants (*Camponotus floridanus, n = 15,133*, *Harpegnathos saltator, n = 15,029*), 3 bees (*Apis mellifera n = 10,145*, *Bombus terrestris n = 9492*, *B. impatiens n = 9869*), *Drosophila melanogaster (r5.30, n = 14,289)*, pea aphid (*Acyrthosiphon pisum* r2, *n* = 38,440), *Tribolium castaneum (v3, 2008, n = 16,985)*, *Daphnia pulex (v1 2007, n = 30,506)*, and human (UniProt 2011, *n* = 20,238). These proteins were aligned using tBLASTn (NCBI) to the repeat and transposon soft-masked genome, then refined with Exonerate [82] to create protein gene models, with options “exonerate --model protein2genome:bestfit --exhaustive 1 --subopt 0 --forcegtag 1 --softmasktarget 1”.

### Gene construction on genome assembly

We constructed OGS2 gene models upon the Nvit_1.0 draft genome assembly, which is the same assembly used for OGS1.2 [4] primarily to preserve tiling array locations. An updated 2.0 genome assembly is also available from the NCBI (NCBI *Nasonia vitripennis* Annotation Release 101), yet does not differ from Nvit_1.0 but for a modest splitting of the largest scaffold into two units and mapping of scaffolds onto the linkage map of *Nasonia* [83]. Transposon and repeat locations remain as found in the initial report, though we performed an updated Repbase database [84] and RepeatMasker run [85] including an evidence quality assessment. OGS1.2 gene models are retained as inputs for our updated version. These lack UTRs for 70 % of genes – a desired improvement. We used NCBI-11 models for *Nasonia* and the published genome assemblies of the two sibling species, *N. longicornis* and *N. giraulti* to assess gene models.

The new *Nasonia* gene models are derived using the evidence-directed AUGUSTUS predictor [86–88]. Several gene prediction sets are produced to create a superset of models that include the models selected to be best, based on matching all gene evidence using EvidentialGene methods [29, 34]. AUGUSTUS flexibly uses both Hidden Markov Model (HMM) training models and available gene evidence for each locus. Training the predictor HMM involves steps described by the authors [87, 88], with validated genes for this species.

We selected 2000 *Nasonia* reference genes that appeared to be full length from the EST/RNA transcript assemblies. We split these into subsets for training and validation of the resulting predictor. We created and used several training sets, plus one that is un-optimized. Evidence sets and configuration weightings were constructed to include: *(1)* complete gene structure information (exon, CDS, intron, gene spans); and *(2)* an extra influence of one major component (proteins, EST exons, full transcript assemblies). The first was necessary to reduce aberrant gene models generated by over-influence of one structure component. For example, evidence of exons from only ESTs or tiling TARs lead to missed introns and missed gene ends. The second was required to reduce conflicting signals, and returned better models under the influence of an appropriate gene evidence class. For instance, extra influence of homologous proteins returned models that more closely matched those proteins. Following each prediction run, the results were assessed for overall quality and matched to evidence. This assessment then suggested the options for new configurations and evidence mixtures. AUGUSTUS is also able to model alternate transcripts from evidence. But those are seldom supported by transcript assemblies and tend to include aberrations. Therefore, we did not use this option and instead used only transcripts assembled directly from EST/RNA reads in selecting alternate splice-forms. We also used as gene information, but not as evidence for re-constructing genes, the version OGS1.2 gene set [4], and NCBI (NCBI-11, RefSeq release v2, September 2011 [38]) gene models for *Nasonia*.

We obtained a total of 333,121 alternate gene models from different evidence sets and parameters, as input to the EvidentialGene classifier (255,785 models from 16 separate AUGUSTUS runs as described above; 18,941 from OGS1.2; 30,379 from EST/RNA assemblies). EvidentialGene uses gene evidence described above from expression and protein sources to annotate each model and exon, then calculates quality scores per model for each type of gene evidence (see next paragraph below). Locus overlaps of gene models are also calculated, using the primary criteria of CDS-overlap on same DNA strand (reverse-strand CDS-overlap is rare, but locus UTR overlaps are relatively common). A weighted sum of the various evidence component scores is calculated, configurable to gene set requirements. Selecting the best locus from among a large set of gene models is accomplished according to two basic criteria: (1) gene evidence must pass a minimum threshold score, and (2) the combined score is maximal for all models overlapping the same CDS-locations. Other criteria and tests are included and used for classification, such as orthology scores. One indicator of a joined model error (Additional file 1: Figure S3) is a homology score for the joined model that is no greater than for un-joined models, though its coding span is larger. Determining a final gene set is an iterative process that involves evaluation after selection, modification of score weights, and reselection. After the majority of optimal models are found, smaller subsets of problem loci are sampled and examined, with additional evaluations to resolve these. This is a negative-feedback process designed to filter out errors and suboptimal gene models, with successive iterations changing fewer models until the optimal set is found. It also involves expert curation to identify and remove suboptimal models, and locate or promote missed high value models (e.g., unique orthologs).

The quality scores per model are calculated using the following types of evidence: *(a)* the level of RNA sequence coverage and tiling array signal over the gene model coordinates on the genome assembly; *(b)* the number of EST and RNA sequence reads spanning the intron splice sites that matched to annotated exon ends; *(c)* gene structure agreement, as end-to-end match of exons in the model with the evidence in support of gene structure, summarized in Table 3 for evidence structure from EST/RNA assemblies and reference proteins; *(d)* sequence homology to proteins from eleven species-specific reference databases using BLASTp scores of all significant matches to the reference set of genes including the number of reference protein matches, bitscore per protein match, and the similarity scores for alignments to same species paralog proteins. These quality scores are summarized for several *Nasonia* gene sets (Table 3) and partitioned according to the source of evidence (EST, RNA sequences, tiled expression spans, reference sequences (*Nasonia* RefSeq), and reference species proteins. Each gene model for each locus is therefore scored by weighted evidence. Finally, the maximal evidence scored, non-overlapping model set is determined, with respect to inter-locus effects of gene joins and other factors.

The EvidentialGene script “annotate_predictions.pl” encapsulates this algorithm. The configurations for this *Nasonia* annotation project are specified in “evigene_wasp2.conf”, which identifies the sources of gene evidence, the gene model sets, the evidence scoring and weighting schemes, plus other factors. An independent evaluation of gene sets for evidence-based recovery is produced by the script “evaluate_predictions.pl”. The summary output table from “evaluate_predictions.pl”, which lists the types of evidence and the recovered gene set, is the source of Table 3. This evidence-based recovery process is calculated for each iterative gene selection, followed by expert examination of sample loci, for adjustments that are made to the weighting scheme, to optimize as many of the evidence components as possible. During this process, the expert-selected models are retained. This evidence scoring of genes is roughly similar to EvidenceModeler [89] and GLEAN [90]. As with EvidenceModeler, an evidence weighing statement is part of the configuration, and an optimal weighting is derived by iterative trials and evaluations.

Coding potential for the gene models was scored according to *(i)* homology to reference proteins, *(ii)* size of calculated open reading frames (ORF in base pairs), *(iii)* relative size of ORF to total transcript size, *(iv)* introns in coding span. These and other measures are commonly used (e.g., [47, 91]), but are often not definitive (see Additional file 10). Our assignment of the gene models to locus type – including protein-coding, non-coding, and transposon – is based on coding potential and other factors that are shared with the NCBI locus typing [38]. For transposons, this includes sequence similarity to known transposon sequences that are previously reported [4], and protein homology to other annotated transposon proteins.

Gene names in OGS2 have been assigned on the basis of sequence alignment to UniProt proteins, to reference insect genes, and to the consensus gene family names from OrthoMCL orthology analyses, by using a BLASTp e-value threshold < 1e-5 and three levels of percentage alignment criteria: levels > 10 % (minimum score to name), > 33 %, and > 66 %. The names are in accordance with UniProt protein naming guidelines [92]. Weak and modest alignments were given the added name qualifiers (“-like” for < 33 %, and “putative” for < 66 %). Some genes were named despite having < 10 % alignment (82); most are transposons with additional evidence of transposon sequence alignment, some are expert choices (e.g., Nasvi2EG008578t1, odorant receptor), and some are poorly associated names. The gene annotations include preferred name, orthology family name, and naming reference gene IDs, and alignment scores.

### Ortholog group assignments and gene family expansions

Orthology of *Nasonia* protein coding genes was assigned using two methods: OrthoMCL [93] and OrthoDB [32]. OrthoMCL was used during gene construction as an essential measure of gene quality, for refining gene model classifications. For OrthoMCL, related species proteomes with *Nasonia* gene models were aligned using all-by-all reciprocal best BLASTp [94, 95] of 11 species’ proteomes (wasp plus those listed above). Alternate transcripts were removed after BLASTp matching, in order to use the most similar gene variants. Clustering of these blast alignments into gene families was also done using OrthoMCL. The resulting gene families are narrow or broad, depending on the chosen alignment options, especially the distance at which to break groups. Resulting groups are rather like the leaves at the tips of a phylogenetic tree. Further MCL clustering of these groups showed relations between many of the narrowly clustered groups. Significance criteria were applied using recommended options: a similarity *p*-value < 1e-05, protein percent identity > 40 %, and MCL inflation of 1.5 (this affects the granularity of clustering). Reciprocal best similarity pairs between species, and reciprocal better similarity pairs within species (i.e., recently arisen paralogs, or in-paralogs, proteins that are more similar to each other within one species than to any protein in the other species) were added to a similarity matrix. The protein similarity matrix was normalized by species and subjected to Markov clustering (MCL; [96, 97]) to generate ortholog groups including recent in-paralogs. An additional round of MCL clustering was applied to identify between-group relations.

After producing the *Nasonia* OGS2 genes, its protein sequences were incorporated into release-6 of the OrthoDB database [32]. Ortholog groups are here defined as groups of genes related by descent from a single common ancestor at the base of the taxonomic level of interest. All genes within a single ortholog group evolved from a series of speciation and/or gene duplication events from a unique ancestor. Their amino acid sequences can thus be aligned and compared with each other. Ortholog groups provide efficient units of analysis for genes over long timescales as they enable partitioning in evolutionarily relevant categories without the need to resolve precise 1 to 1 relationships. From the total 24,388 OGS2 genes, 15,173 (62 %) could be assigned to an ortholog group among the Arthropoda in OrthoDB version 6.

We assessed which ortholog groups are characterized by evolutionary expansions in the *Nasonia* lineage. We selected 9601 ortholog groups that have paralogs in *Nasonia* and over 80 % of the other sequenced Arthropoda. To further increase the stringency of the selection criteria, we removed all genes from this set that have any duplicates in other hymenopteran species. Of the total 9601 ortholog groups, 411 (0.05 %) have duplicates specific to the *Nasonia* lineage among the Hymenoptera. We used sequence similarity searches to cross-validate the absence of ultra-conserved ortholog groups of the BUSCO dataset (OrthoDB) from the *Nasonia* genome. We retrieved protein sequences for all genes within those ortholog groups from all sequenced arthropods.

### Identification of fast- and slow-diverging genes in the *Nasonia* relative to ants and bees

We retrieved amino-acid alignments for ortholog groups among the Hymenoptera from OrthoDB version 6 and selected those that contained at least one gene in the *Nasonia* genome and at least one gene in one ant and one bee genome (8696 OGs). We generated a pairwise sequence divergence matrix, comparing all genes versus all genes within each of those ortholog groups by applying a JTT protein evolution model as implemented in the R package phangorn [98]. We then estimated the proportion of between-genus sequence divergence due to the *Nasonia* genes using the following ratio

$$ \frac{AN+ BN}{AN+ BN+ AB} $$

where AN and BN are the median pairwise amino-acid distances between the *Nasonia* gene and Ant or Bee orthologs respectively, and AB is the median pairwise distance between the ant and bee orthologs in the genes’ ortholog group. We analyzed this ratio with a generalized linear mixed model (GLMM) with logit link function, using overall median sequence divergence of the ortholog group, presence of *Nasonia* paralogs and transposon-associated expression as predictors to account for the role of those factors in protein evolution. We also used the ortholog group ID as a random blocking factor to account for individual differences in evolutionary rates between ortholog groups. We then extracted the GLMM’s residuals to evaluate the remaining unexplained levels of sequence evolution. We selected genes that exceeded the 95th percentile of the distribution of residuals as highly diverging, and those below the 5th percentile as slowly diverging. We did not include relative non-synonymous to synonymous substitution rates in the GLMM because the analysis is based on protein sequence alignments scored by a weighted matrix of amino acid substitutions.

To avoid false positives due to exceedingly fast or slow protein sequence evolution in either the ant or bee clade, we also computed separately the rates of divergence between *Nasonia* and the ant or bee lineages (AN/AN+BN+AB and BN/AN+BN+AB). We then generated two independent GLMMs for these ratios with the same factors used for the compound ratio and reported the genes that scored as significantly faster or slower (above 80th percentile or below 20th percentile) in both cases. This second set provides a high confidence list of genes that are differentially diverging in the *Nasonia* lineage but show limited differentiation between the ant and bee lineages. We point out that this is a tool to identify proteins that may be evolving more quickly at the amino acid level in the *Nasonia* clade. Because the analysis is unrooted, the method does not identify proteins that are specifically evolving more quickly since divergence of *Nasonia* from its common ancestor with ants and bees, but also includes changes from that common ancestor to the split between ants and bees. More precise evolutionary analyses will require phylogenetic reconstruction for all the genes, but the current set is useful for identifying likely candidates for divergence among these taxa. Given the very long branches involved in such analyses, use of dN/dS ratios as an index of adaptive evolution would be inappropriate due to total saturation of synonymous substitutions.

### Functional enrichment testing

We tested all gene sets for functional enrichment of Gene Ontology (GO) terms obtained by Blast2GO [99], using the two-tailed Fisher’s exact test with a False Discovery Rate (FDR) of 5 % against the complete gene complement of *N. vitripennis*. The *Nasonia* GO annotation for OGS2 was provided by the *Nasonia* community [70]. Of the 24,388 OGS2 genes with supporting evidence, 24,373 are present in the community-provided Blast2Go annotation files and 6446 of these (26,4 %) have GO assignments.

### Alternative splicing analysis

We used GLMMs to test for factors correlated with the presence or absence of alternative transcripts in OGS2. Our test factors include presence of strict sense paralogs (defined as reciprocal best sequence similarity match within the same genome versus reciprocal best match within other genomes), number of broad sense paralogs (genes within the same genome belonging to the same arthropod OrthoDB ortholog group plus one, log and z transformed), number of predicted introns (log and z transformed), transcript length (log and z transformed, using the longest transcript per gene), proportion of coding sequence over total transcript length (CDS/Transcript length, log transformed and normalized), ratio of *Nasonia*-specific protein evolution (see Methods section “Identification of fast- and slow-diverging genes in the *Nasonia* relative to ants and bees”, log and z transformed), methylation status in adult females [16] and phylostratigraphic age [15].

We selected only genes with a complete record for all tested factors. Since the detection of isoforms is proportional to the coverage of that gene, we further restricted our analyses only to genes with both strong expression support and strong intron support, which have comparable levels of transcriptional data available. Therefore, our final dataset was comprised of 5447 genes. To estimate over-dispersion, we fitted a GLM with quasi-binomial error distribution including all analysis parameters. This model did not show over-dispersion, with a c-hat of 1. We therefore fitted subsequent models to a binomial distribution with logit link function. All subsequent models also included a random intercept error structure for each ortholog group among arthropods, to account for different selective pressure on different gene families.

We estimated the support of individual factors by fitting a full model incorporating all parameters, then compared this model to others incorporating all factor combinations by applying the Akaike Information Criterion, corrected for finite sample size (AICc). We calculated the relative importance of factors as the sum of weights of all models containing that factor over the total weight of all models within the set. Since the final model set contained several models with similar AICc values (Additional file 13), we choose to present the results as model-averaged estimates rather than to choose a single best model.

### Mapping OGS2 to Nvit_2.1 reference genome assembly

To map GFF files between assemblies, we first generated a chain file as follows: we split the Nvit_1.0 assembly into 5 kb fragments, and aligned each fragment to the Nvit_2.1 reference using BLAT (options: tileSize = 11, minScore = 100, minIdentity = 98, fastMap), using an ooc file produced with the makeOoc option to BLAT. We then combined all the BLAT output using liftUp to convert the result files into the parent (in this case Nvit_1.0) coordinate system. The resulting psl file was processed with axtChain (options: linearGap = medium, psl), chainMergeSort, chainSplit (options: lump = 20), chainNet, and chainSubset to produce a chain file. We then produced the reciprocal file using chainSwap. Both chain files (Nvit_to NVIT, and Nvit_to NVIT) are provided as supplemental material (Additional file 5).

### Additional software tools

Most statistical analyses were performed in R version 3.0.0 [100] using the following packages: plyr [101] and reshape2 [102] for data handling, phangorn for sequence analyses [98], lme4 [103] for GLMMs, MuMIn [104] for multi-model comparisons and model-averaging, vcd [105] and ggplot2 [106] for plotting. Functional enrichment testing was performed using Blast2GO [99].

## Abbreviations

BUSCO, benchmarking universal single copy orthologs; CDS, protein coding sequences; EST, expressed sequence tags; GLMM, generalized linear mixed model; lncRNA, long noncoding RNA; mRNA, messenger RNA; NCBI-101, NCBI *Nasonia vitripennis* annotation release 101; Nvit_1.0, *Nasonia vitripennis* genome assembly 1.0; Nvit_2.1, *Nasonia vitripennis* genome assembly 2.1; OG, orthologous group; OGS1.2, official gene set 1.2; OGS2, official gene set 2; RNA-Seq, RNA-sequencing; UTR, untranslated region

## Additional files

Additional file 1: Figure S1. Expression values relative to gene structures for RNA-Seq (Reads) and genome tiling path microarrays (Tile) for species *Nasonia* (purple, this project), *Drosophila* (red, blue, [74]) and *Daphnia* (green, [107]). Annotated gene near-exon spans are scored per base for average expression scores from the data sets, and relative expression plotted with respect to gene transcript start (first exon), stop (last exon), and inner exon start, stop positions. Both methods (genome tiling and RNA-Seq) have abrupt expression strength changes at exon boundaries, on average, indicating their value in modeling gene structure positions. Expression scores are read-coverage for RNA-Seq, and log-normalized intensity for tiling array, as described in the Methods section. **Figure S2.** Gene modeling example with tile expression data, including gene evidence (upper tracks with tiling, introns, proteins), tiling TAR-exon to Exonerate models (middle), and gene predictions from tile TAR hints (lower), on genome map. The lower tracks have excessive false UTR spans attached to gene models, primarily due to tiling expression that lacks gene start/stop and intron splice joining signals. These false UTR spans are supported by expression evidence, but as a combination of alternate exons, separate gene loci, and non-coding expression. Intermediate tracks (Exonerate models) often match gene structures from other methods, but have a high proportion of unsupported exon extensions as for lower track. **Figure S3.** Gene join error example. A mistaken gene model from honey bee (tan, lower, LOC552483) is transferred to *Nasonia* in NCBI RefSeq models (dark orange, middle), merging a ribosomal protein (right) and Ankyrin repeat protein (left). EvidentialGene models (yellow, top) did not contain this mistake, due to the combination of RNA-Seq assemblies (purple, bottom) that are un-joined (but could be parts of one gene), the lack of intron joining evidence, and the orthology assessment metrics that distinguish gene joins from true complete genes. NCBI Refseq models for both *Apis* (new LOC102654426 and mRpL52 in NCBI *Apis* rel. 102) and *Nasonia* have been updated to correct this join error. **Figure S4.** Log counts of methylated and unmethylated genes in different classes of expression support. Grey bars indicate genes with no known methylation status. (ZIP 938 kb) Additional file 2: Table S1. Consensus in the location of the OGS2 gene set on the genome assemblies of sibling species *Nasonia longicornis* and *N. giraulti*, including recent, high identity paralogs. Almost all OGS2 genes are located on 2 sibling species draft assemblies [4], using GMAP [36] transcript mapping. Paralog locus consensus patterns are tabulated for inparalogs (sharing orthology to other species) and uniquepar (lacking strong homology to other species). Of the total paralog families, each with several genes, most paralogs are on different scaffolds for all species. The counts of tandem paralogs with different separations are indicated. **Table S2.** A set of 62 orthology groups found in *Nasonia* transcript assemblies that are poorly mapped onto the current genome, but should be considered as part of a complete *Nasonia* gene set. **Table S3.** A total of 75 orthology groups missing from *Nasonia* but found in 9 other insect genomes. **Table S4.** Histone genes present in OGS2.0 annotated with presence or absence of lineage-specific expansions. NA entries were not assigned to orthologous groups at the level of Hymenoptera. (ZIP 33 kb) Additional file 3: OrthoDB6 BUSCO (Benchmarking Universal Single Copy Orthologs) genes missing from OGS2. (XLS 1367 kb) Additional file 4: OrthoDB6 BUSCO (Benchmarking Universal Single Copy Orthologs) genes present in multiple copies in OGS2. (XLS 51 kb) Additional file 5: Chain files, GFF mapping and transcript status of OGS2 on Nvit_2.1 genome assembly. (XLS 17233 kb) Additional file 6: Table of OGS2 gene transcripts equivalences to OGS1 and NCBI-101 gene sets, using the CDS-exon locations on the genome assembly. “NCBI101geneID” and “OGS1geneID” include equivalence value as percent equal to CDS.EXON. For example: Nasvi2EG000002t1 nasvn14g1803t1/99.70 is 99 % CDS equal, 70 % exon equal; NV10001-RA/74.89 is 74 % CDS equal, 89 % exon equal. The “NCBI101geneID” is the local ID “gene1803” from the NCBI GFF gene table, adding “nasvn14g” prefix and alternate transcript suffix “t1,t2,..”, with associated column of public “NCBI101transcriptID” (XM_ or NM_). “Genome21loc” is the gene span location on Nvit_2.1 scaffold assembly, “Genome1loc” is the gene span location on Nvit_1.0 scaffold assembly (most Nvit_2.1 and 1.0 are equivalent). “NCBI101also” and “OGS1also” are additional gene transcripts with partial equivalence to the OGS2 gene. Nonequivalence values: “na” for “NCBI101geneID”, “novid” for “OGS1geneID”. “WaspAtlasNCBI101” lists NCBI-101 equivalent genes provided by [40]. Disagreements are marked by “*”. These appear to be UTR-exon or gene-span overlaps, rather than the CDS-exon overlap used in this table equivalence. (XLSX 2889 kb) Additional file 7: OGS2 genes whose ortholog groups are characterized by lineage-specific expansions or contractions. (XLS 3711 kb) Additional file 8: Protein evolutionary distances of OGS2.0 genes compared to ant and bee lineages, residuals distances after model fitting and fast/slow evolving categorization at the 5th and 20th quantile threshold. (XLS 2790 kb) Additional file 9: Protein alignment of the OG EOG6R4ZDK (hymenopteran histone H3). Clipped to include only residues shared between all genes. (TXT 13 kb) Additional file 10: Supplement document on long non-coding RNA expression and genes within the *Nasonia* genome and genomes of other animals. (DOCX 6088 kb) Additional file 11: Genes with more than 10 isoforms present in OGS2. (XLS 48 kb) Additional file 12: Figure S5. Correlation between methylation status and expression support in OGS2.0. (PDF 4 kb) Additional file 13: Model selection table for models comprising different combinations of factors with a putative role in characterizing genes with and without annotated isoforms. (XLS 59 kb)
