# Supplementary figures and images for: OGS2: genome re-annotation of the jewel wasp Nasonia vitripennis
Source: BMC Genomics. 2016 Aug 25;17(1):678. doi: 10.1186/s12864-016-2886-9 (PMC5000498; doi:10.1186/s12864-016-2886-9)

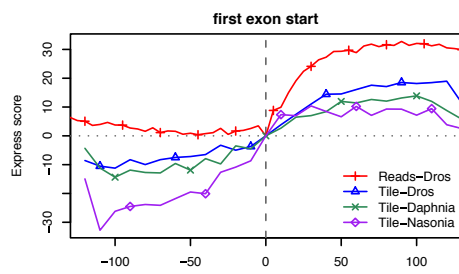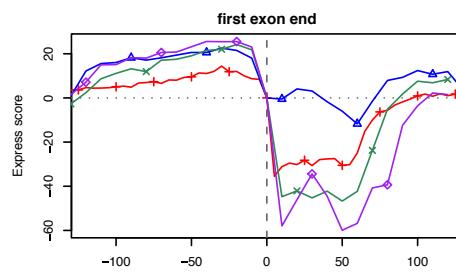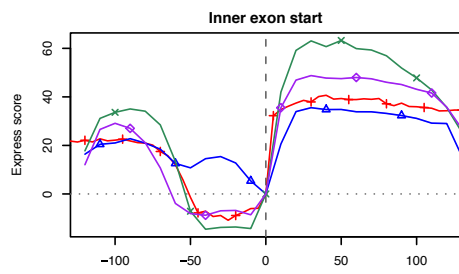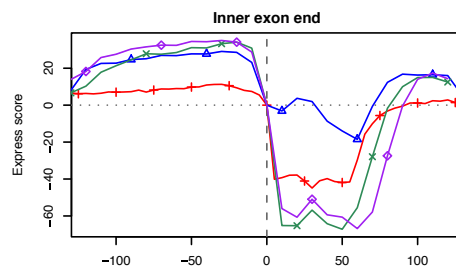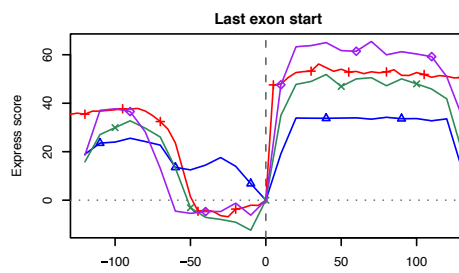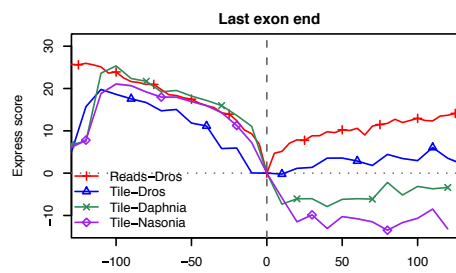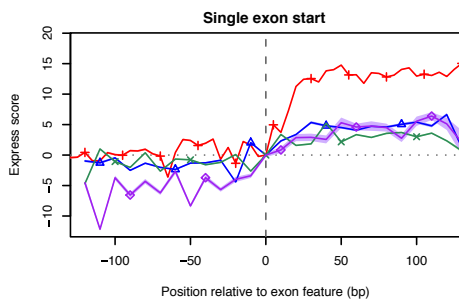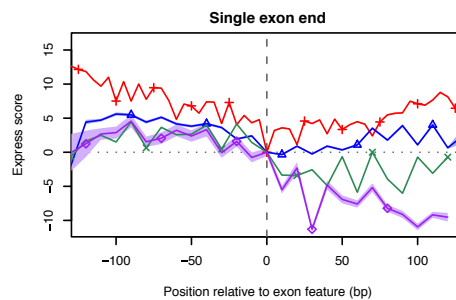

Supplement: Additional file 1: Figure S1. — Expression values relative to gene structures for RNA-Seq (Reads) and genome tiling path microarrays (Tile) for species Nasonia (purple, this project), Drosophila (red, blue, [74]) and Daphnia (green, [107]). Annotated gene near-exon spans are scored per base for average expression scores from the data sets, and relative expression plotted with respect to gene transcript start (first exon), stop (last exon), and inner exon start, stop positions. Both methods (genome tiling and RNA-Seq) have abrupt expression strength changes at exon boundaries, on average, indicating their value in modeling gene structure positions. Expression scores are read-coverage for RNA-Seq, and log-normalized intensity for tiling array, as described in the Methods section. Figure S2. Gene modeling example with tile expression data, including gene evidence (upper tracks with tiling, introns, proteins), tiling TAR-exon to Exonerate models (middle), and gene predictions from tile TAR hints (lower), on genome map. The lower tracks have excessive false UTR spans attached to gene models, primarily due to tiling expression that lacks gene start/stop and intron splice joining signals. These false UTR spans are supported by expression evidence, but as a combination of alternate exons, separate gene loci, and non-coding expression. Intermediate tracks (Exonerate models) often match gene structures from other methods, but have a high proportion of unsupported exon extensions as for lower track. Figure S3. Gene join error example. A mistaken gene model from honey bee (tan, lower, LOC552483) is transferred to Nasonia in NCBI RefSeq models (dark orange, middle), merging a ribosomal protein (right) and Ankyrin repeat protein (left). EvidentialGene models (yellow, top) did not contain this mistake, due to the combination of RNA-Seq assemblies (purple, bottom) that are un-joined (but could be parts of one gene), the lack of intron joining evidence, and the orthology assessment metrics that distinguish gene jo [file 12864_2016_2886_MOESM1_ESM.zip › 12864_2016_2886_MOESM1_ESM/Figure S1.pdf]

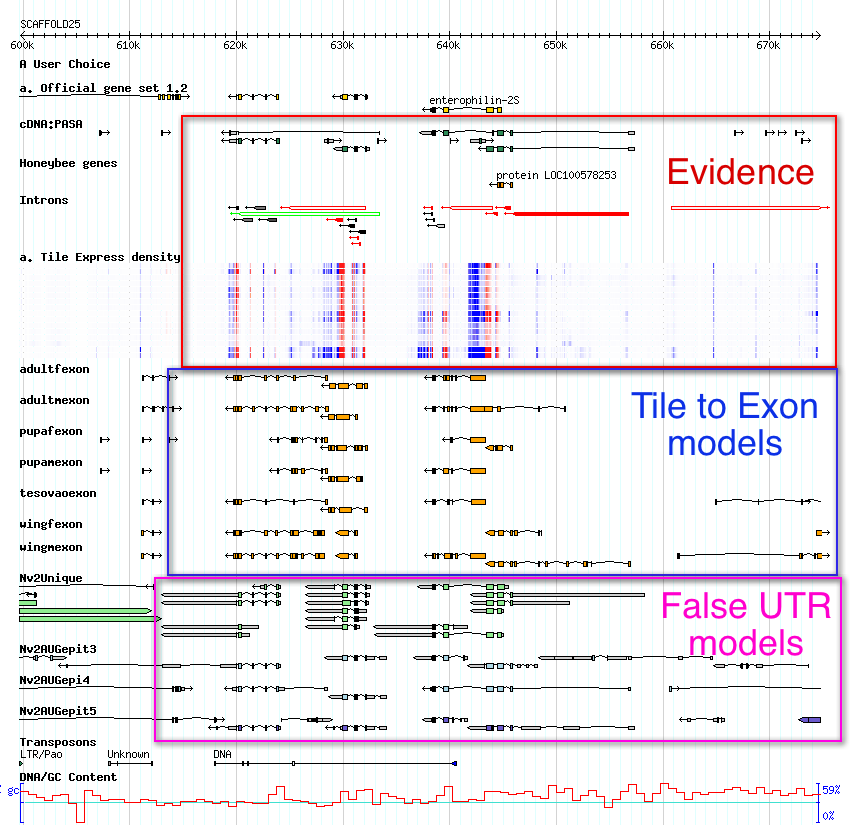

Supplement: Additional file 1: Figure S1. — Expression values relative to gene structures for RNA-Seq (Reads) and genome tiling path microarrays (Tile) for species Nasonia (purple, this project), Drosophila (red, blue, [74]) and Daphnia (green, [107]). Annotated gene near-exon spans are scored per base for average expression scores from the data sets, and relative expression plotted with respect to gene transcript start (first exon), stop (last exon), and inner exon start, stop positions. Both methods (genome tiling and RNA-Seq) have abrupt expression strength changes at exon boundaries, on average, indicating their value in modeling gene structure positions. Expression scores are read-coverage for RNA-Seq, and log-normalized intensity for tiling array, as described in the Methods section. Figure S2. Gene modeling example with tile expression data, including gene evidence (upper tracks with tiling, introns, proteins), tiling TAR-exon to Exonerate models (middle), and gene predictions from tile TAR hints (lower), on genome map. The lower tracks have excessive false UTR spans attached to gene models, primarily due to tiling expression that lacks gene start/stop and intron splice joining signals. These false UTR spans are supported by expression evidence, but as a combination of alternate exons, separate gene loci, and non-coding expression. Intermediate tracks (Exonerate models) often match gene structures from other methods, but have a high proportion of unsupported exon extensions as for lower track. Figure S3. Gene join error example. A mistaken gene model from honey bee (tan, lower, LOC552483) is transferred to Nasonia in NCBI RefSeq models (dark orange, middle), merging a ribosomal protein (right) and Ankyrin repeat protein (left). EvidentialGene models (yellow, top) did not contain this mistake, due to the combination of RNA-Seq assemblies (purple, bottom) that are un-joined (but could be parts of one gene), the lack of intron joining evidence, and the orthology assessment metrics that distinguish gene jo [file 12864_2016_2886_MOESM1_ESM.zip › 12864_2016_2886_MOESM1_ESM/Figure S2.png]

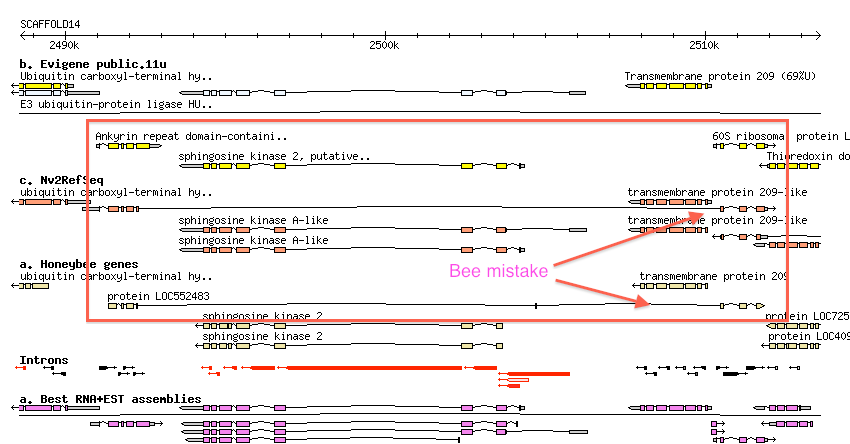

Supplement: Additional file 1: Figure S1. — Expression values relative to gene structures for RNA-Seq (Reads) and genome tiling path microarrays (Tile) for species Nasonia (purple, this project), Drosophila (red, blue, [74]) and Daphnia (green, [107]). Annotated gene near-exon spans are scored per base for average expression scores from the data sets, and relative expression plotted with respect to gene transcript start (first exon), stop (last exon), and inner exon start, stop positions. Both methods (genome tiling and RNA-Seq) have abrupt expression strength changes at exon boundaries, on average, indicating their value in modeling gene structure positions. Expression scores are read-coverage for RNA-Seq, and log-normalized intensity for tiling array, as described in the Methods section. Figure S2. Gene modeling example with tile expression data, including gene evidence (upper tracks with tiling, introns, proteins), tiling TAR-exon to Exonerate models (middle), and gene predictions from tile TAR hints (lower), on genome map. The lower tracks have excessive false UTR spans attached to gene models, primarily due to tiling expression that lacks gene start/stop and intron splice joining signals. These false UTR spans are supported by expression evidence, but as a combination of alternate exons, separate gene loci, and non-coding expression. Intermediate tracks (Exonerate models) often match gene structures from other methods, but have a high proportion of unsupported exon extensions as for lower track. Figure S3. Gene join error example. A mistaken gene model from honey bee (tan, lower, LOC552483) is transferred to Nasonia in NCBI RefSeq models (dark orange, middle), merging a ribosomal protein (right) and Ankyrin repeat protein (left). EvidentialGene models (yellow, top) did not contain this mistake, due to the combination of RNA-Seq assemblies (purple, bottom) that are un-joined (but could be parts of one gene), the lack of intron joining evidence, and the orthology assessment metrics that distinguish gene jo [file 12864_2016_2886_MOESM1_ESM.zip › 12864_2016_2886_MOESM1_ESM/Figure S3.png]

**A**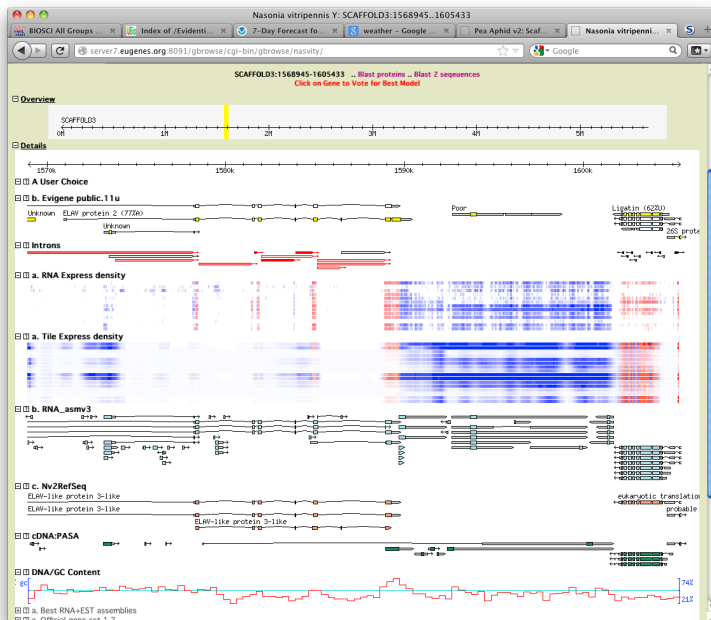**B**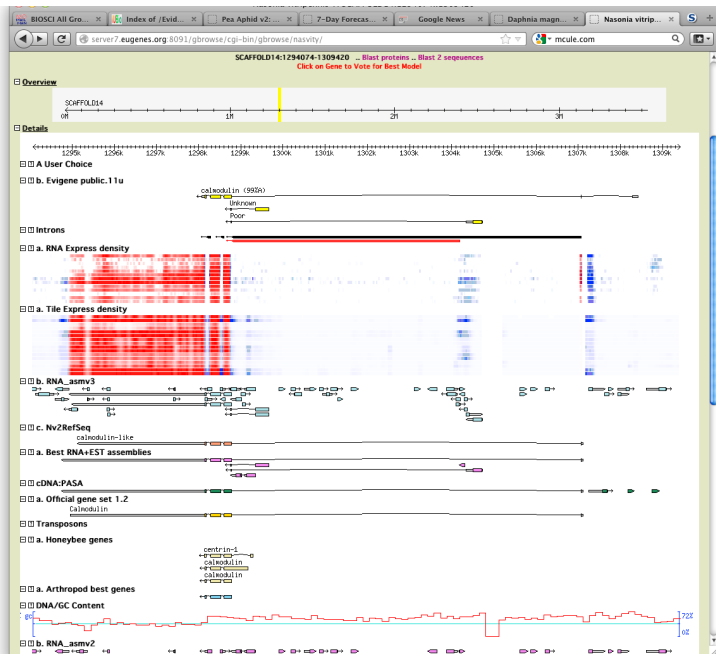**C**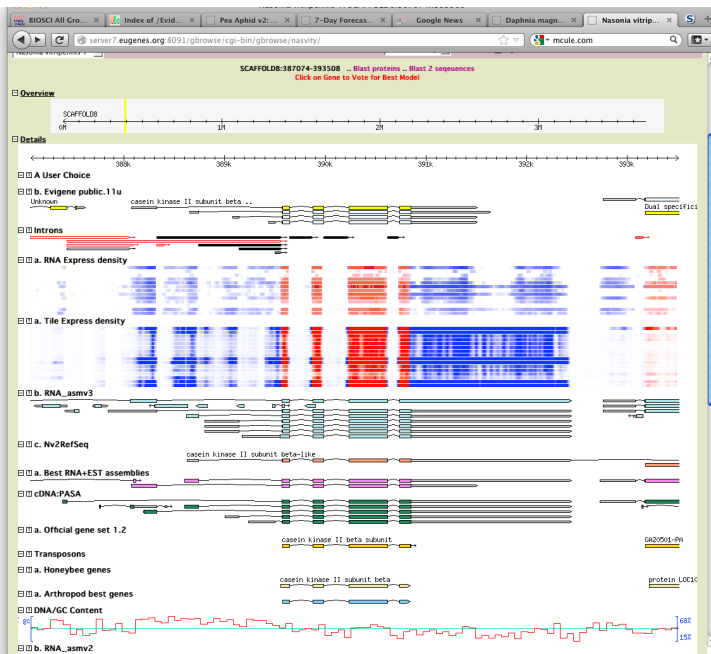**D**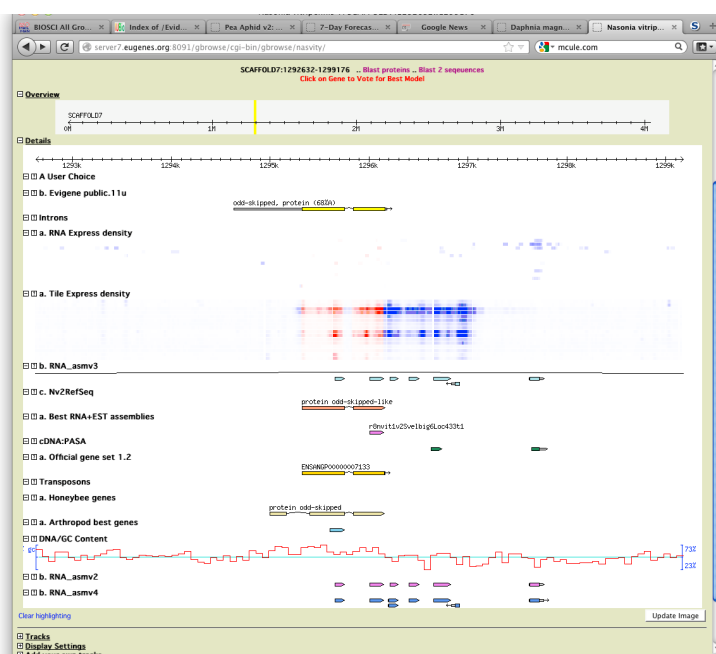**E**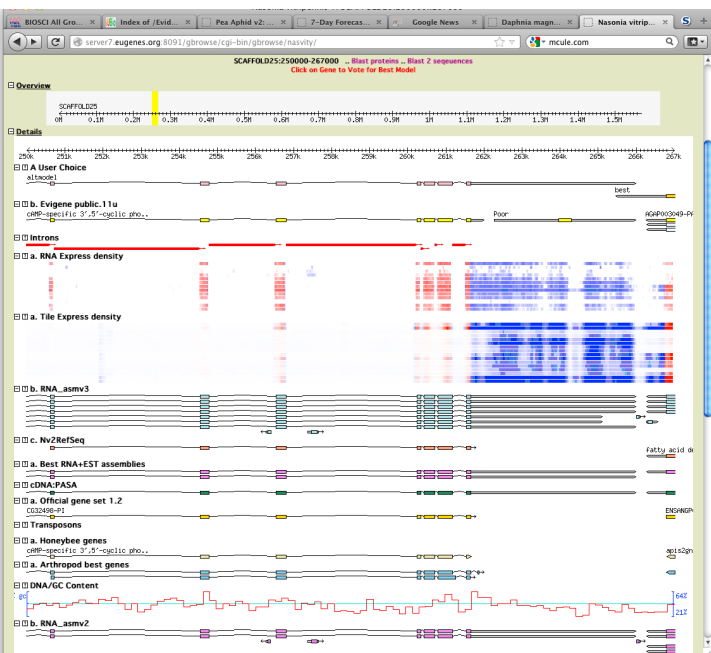**F**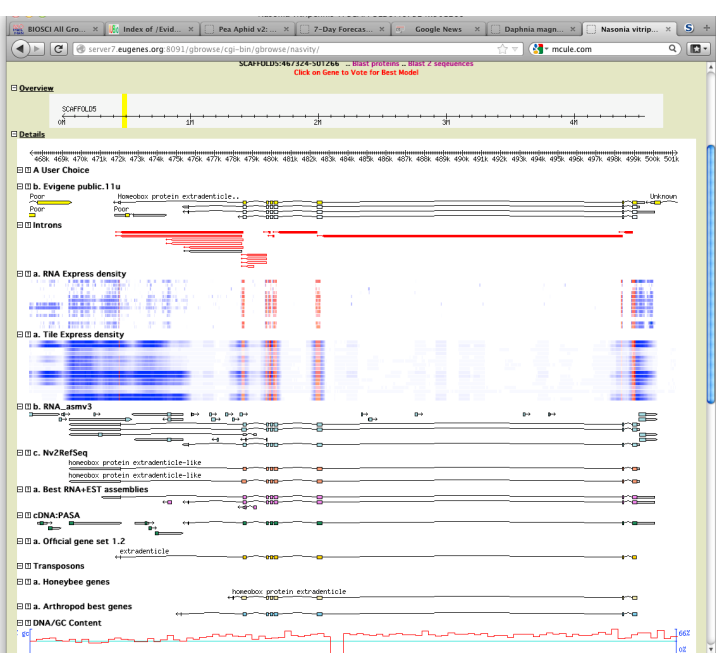

Supplement: Additional file 1: Figure S1. — Expression values relative to gene structures for RNA-Seq (Reads) and genome tiling path microarrays (Tile) for species Nasonia (purple, this project), Drosophila (red, blue, [74]) and Daphnia (green, [107]). Annotated gene near-exon spans are scored per base for average expression scores from the data sets, and relative expression plotted with respect to gene transcript start (first exon), stop (last exon), and inner exon start, stop positions. Both methods (genome tiling and RNA-Seq) have abrupt expression strength changes at exon boundaries, on average, indicating their value in modeling gene structure positions. Expression scores are read-coverage for RNA-Seq, and log-normalized intensity for tiling array, as described in the Methods section. Figure S2. Gene modeling example with tile expression data, including gene evidence (upper tracks with tiling, introns, proteins), tiling TAR-exon to Exonerate models (middle), and gene predictions from tile TAR hints (lower), on genome map. The lower tracks have excessive false UTR spans attached to gene models, primarily due to tiling expression that lacks gene start/stop and intron splice joining signals. These false UTR spans are supported by expression evidence, but as a combination of alternate exons, separate gene loci, and non-coding expression. Intermediate tracks (Exonerate models) often match gene structures from other methods, but have a high proportion of unsupported exon extensions as for lower track. Figure S3. Gene join error example. A mistaken gene model from honey bee (tan, lower, LOC552483) is transferred to Nasonia in NCBI RefSeq models (dark orange, middle), merging a ribosomal protein (right) and Ankyrin repeat protein (left). EvidentialGene models (yellow, top) did not contain this mistake, due to the combination of RNA-Seq assemblies (purple, bottom) that are un-joined (but could be parts of one gene), the lack of intron joining evidence, and the orthology assessment metrics that distinguish gene jo [file 12864_2016_2886_MOESM1_ESM.zip › 12864_2016_2886_MOESM1_ESM/Figure S4.pdf]

Correlation between methylation status and expression support in OGS2.0

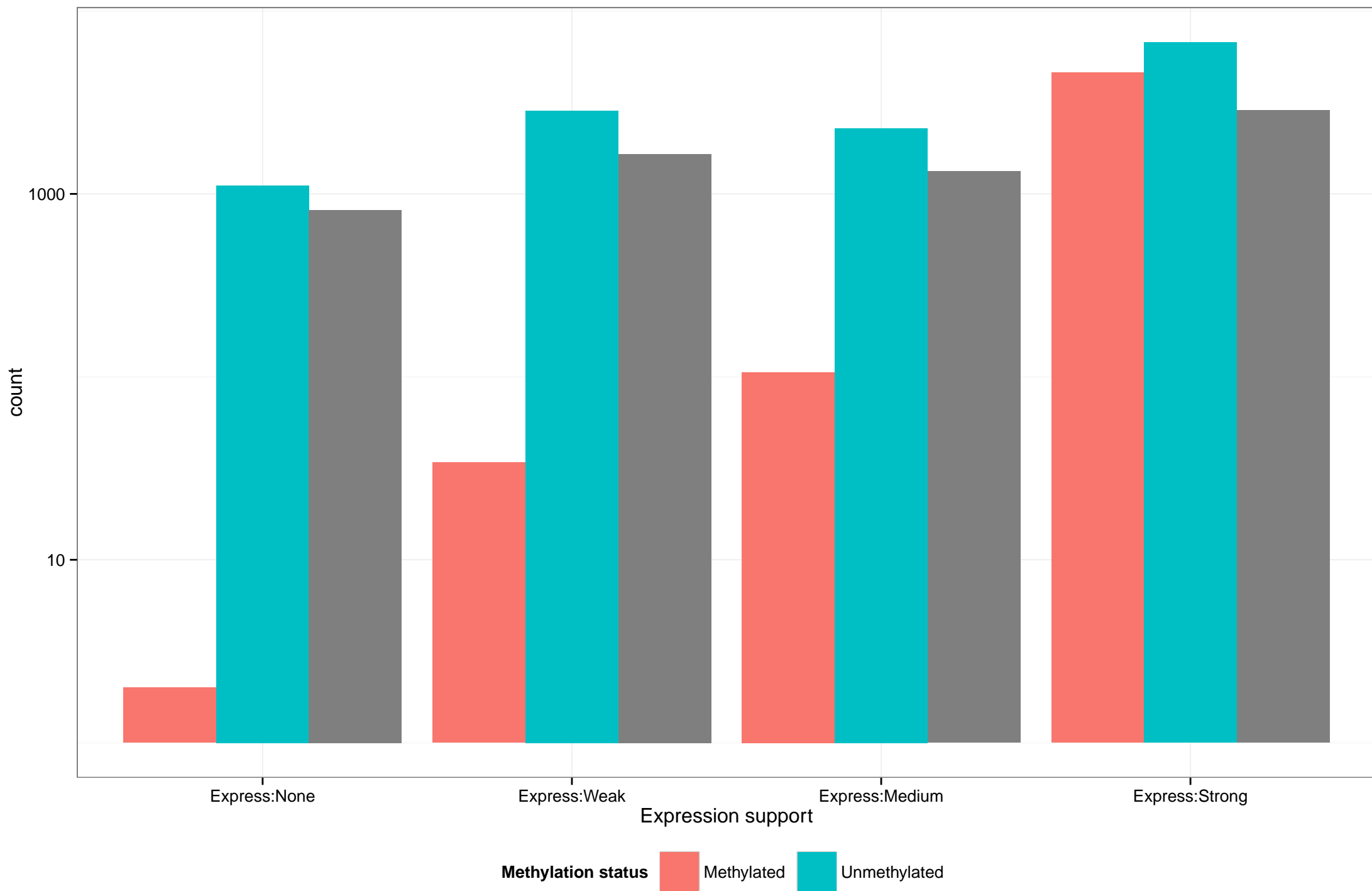

Supplement: Additional file 12: Figure S5. — Correlation between methylation status and expression support in OGS2.0. (PDF 4 kb) [file 12864_2016_2886_MOESM12_ESM.pdf]
