# Supplementary material for: OGS2: genome re-annotation of the jewel wasp Nasonia vitripennis
Source: BMC Genomics. 2016 Aug 25;17(1):678. doi: 10.1186/s12864-016-2886-9 (PMC5000498; doi:10.1186/s12864-016-2886-9)
Supplement: Additional file 10: — Supplement document on long non-coding RNA expression and genes within the Nasonia genome and genomes of other animals. (DOCX 6088 kb) [file 12864_2016_2886_MOESM10_ESM.docx]

**Additional file 10**

**Supplement document on long non-coding RNA expression and genes within the *Nasonia* genome and genomes of other animals**

Below are examples of conserved protein coding genes with highly expressed, long, non-coding sequences located adjacent to 3' coding sequence. Some of these also have 5' long non-coding spans that are joined or detached. Both cdsRNA and ncRNA at these locus complexes are conserved across vertebrates and arthropods. The examples below are of orthologous genes with gene-expression evidence maps for mouse, fruit fly, honey bee, harvest ant and jewel wasp. In these examples, ncRNA spans are often longer than cdsRNA spans, have greater expression levels, and are poorly modeled compared to cdsRNA.

Sequence conservation across species is generally low for non-coding RNA. But secondary folding structure and binding site motifs are often more conserved [56]. Positional conservation is another aspect of study for non-coding loci, as these may be found adjacent to conserved coding loci across species [56, 51, 52]. We use this aspect to provide examples of loci that can be compared. Yet we do not measure whether their non-coding expression is conserved.

The RNA-Seq and tiling array evidence is ambiguous, conflicting, and complex on whether these are UTRs of coding gene loci, detached lncRNA loci, or a mixture of both. Some of these elements in the genome are indeed a mixture of both, located at same relative positions in *Drosophila* for *calmodulin*, *extradenticle* plus 13 other coding genes. These ambiguous models are listed within tables from two publications of the *Drosophila* modENCODE project analyzing the same data, listed in Table S4a; coding genes are named and their locations are listed in Table S1. "A Full Lists of CNS-Extended 3′ UTRs" of [50], and the lincRNA genes are listed in "Supplementary Table 1: LincRNA loci, genomic locations, FPKM values ..", from <http://gbe.oxfordjournals.org/content/suppl/>2012/03/01/evs020.DC1/Young_Supplementary_Tables.xls [51].

The expression evidence for the *Drosophila* elements include paired-end RNA-Seq data (35-70 bp reads) plus EST data (500 bp reads), including intron splice sites mapped to the genome. Some of these sequences are strand-specific which, along with splice sites, provide orientation for the transcriptional elements. Ambiguity of transcriptional units arises from conflicts among the data sources, including gaps in areas of high expression not spanned by spliced reads (introns), and anti-sense or reverse transcription. Gaps in expression are confounded with gaps in genome assembly for some, but not all examples shown here.

We use the abbreviation "xco-lncRNA" for these highly expressed, conserved, long non-coding RNA spans of ambiguous structure. Our case studies of xco-lncRNA are: (a) *ELAV-2 RNA-binding protein* (Fig. S4.1), (b) *calmodulin CaMKI* (Fig. S4.2), (c) *casein kinase II beta* or *CkIIbeta* (Fig. S4.3), (d) *odd-skipped* (Fig. S4.4), (e) *dunce/cAMP-specific 3',5'-cyclic phosphodiesterase* (Fig. S4.5), and (f) homeobox protein *extradenticle* or *exd* (Fig. S4.6). Gene and expression maps from the “NCBI Genes” database resource are shown for each of these loci along with the *Nasonia* Evigene gene-evidence maps. NCBI GeneID numbers are given for each case, found at URL <http://www.ncbi.nlm.nih.gov/> gene/{GeneID}, where ant is *Pogonomyrmex barbatus* (red harvest ant), bee is *Apis mellifera*, fly is *Drosophila melanogaster*, mouse is *Mus musculus*. Gene names are of *Drosophila* with variants for mouse (e.g., *ELAV-like-2*). On these figures (S4.1-S4.6), purple ovals indicate the long non-coding expression spans, and purple arrows point to annotated lncRNA loci.

The gene construction methods used for *Nasonia* OGS2 recover some of this xco-lncRNA. These methods are similar to those used for the other species that are shown (ant, bee, fly, mouse) by NCBI [38] and others. Mouse and fly genes and evidence are those modeled and curated by model organism consortia, plus NCBI’s methods for mouse. Ant and bee genes are from NCBI modeling methods, which have annotated 2434 lncRNA in the bee genome, and 930 lncRNA for the ant genome. The annotated lncRNA loci are supported by expression evidence, but have weak coding.

**What is "non-coding"?**

Loci that are classified as non-coding in these gene sets all have some coding potential, and ORFs up to 300 amino acids (aa) are found within them, though most are under 80 aa. NCBI *Apis* lncRNA transcripts all have ORFs ranging in size from 290 aa or less, similar to the *Nasonia* OGS2 non-coding set. NCBI *Apis* and the Evigene *Nasonia* loci are classified either as lncRNA or mRNA depending on measures that include: (i) homology to known proteins or transposons, (ii) non-coding spans longer than coding and relatively short coding spans, (iii) coding potential calculations (e.g. codon usage, [92]), (iv) introns in coding spans, and (v) conservation of coding sequence between species or populations (non-synonymous/synonymous code changes). These measure positive coding attributes that define mRNA, but are ambiguous for an lncRNA classification. An example NCBI lncRNA annotation associated with the *Apis* gene *exd* is listed below (Fig. S4.6).

**What is an "lncRNA" locus or gene?**

We employed the criteria outlined above, although our gene annotations use the term "noncode", which are equivalent criteria to the VEGA “lncRNA” classification [(ht](http://vega.sanger.ac.uk/info/about/)t[p://vega.sanger.ac.uk/info/about/](http://vega.sanger.ac.uk/info/about/) gene_and_transcript_types.html). lncRNA is the general class assignment, which is supplemented by sub-classification terms listed below, where subclasses 2 to 9 may provide further detailed annotations:

1. Non coding: Contains transcripts that are known from the literature to not be protein coding.
2. 3prime_overlapping_ncRNA: Are transcripts where ditag and/or published experimental data strongly support the existence of long (>200bp) non-coding transcripts that overlap the 3'UTR of a protein-coding locus on the same DNA strand.
3. Antisense: Are transcripts that overlap the genomic span (i.e. exon or introns) of a protein-coding locus on the opposite strand.
4. lincRNA (long interspersed ncRNA): Are transcripts of a long intergenic non-coding RNA locus with length >200bp. Requires lack of coding potential and may not be conserved among species.
5. Retained_intron: Are alternatively spliced transcripts believed to contain intronic sequence relative to other, coding, variants.
6. Sense_intronic: Are long non-coding transcripts within introns of a coding gene that do not overlap with any exons.
7. Sense_overlapping: Are long non-coding transcripts that contain a coding gene in its intron on the same strand.
8. Macro_lncRNA: Are un-spliced lncRNAs that are several Kbp in size.
9. Bidirectional lncRNA: Are non-coding loci that originate from within the promoter region of a protein-coding gene, with transcription proceeding in the opposite direction on the opposite strand.

There can be confusion about lncRNA or lincRNA terms, because these are also used by others for a subset of non-coding loci that have been validated by experimental evidence. Our usage is for the superset that has expressed RNA support and computational validations.

cdsRNA and xco-lncRNA gene segments are equivalently modeled using a combination of methods, as described earlier for de-novo assembly of RNA-Seq to transcripts, genome-mapped assemblies (with PASA and Cufflinks for EvidentialGene, Splign for NCBI), and by gene modeling using Hidden Markov Model statistical assessment of structure signals of transcriptional start and stop sites, plus intron splice sites (with AUGUSTUS for EvidentialGene, and GNOMON for NCBI). The expression spans are linked into a single gene model when RNA-Seq read pairs overlap taking into account detected gene structure signals. Yet even though these varying methods utilize the same data as evidence, they often produce different, conflicting, models – especially for non-coding elements where their structures are less definite. A benchmark comparison of gene modeling and reconstruction methods using RNA-Seq data as evidence to reconstruct human and fly non-coding and coding genes [53], emphasizes this discrepancy: only 25% non-coding exons are recovered (still fewer complete lncRNA transcripts) *versus* 75% coding exons recovered on average for several gene modelers and RNA-assemblers, including those methods used in this *Nasonia* project.

Our expert interpretation of the six annotated xco-lncRNA examples shown below is that expression spans appear to be fairly conserved from fly to mouse, but gene models are missing much expression data, while other expressed parts are mis-modeled. Example (Fig. S4.3) *CkIIbeta/casein kinase II beta* is most likely a long UTR of CDS for all species. Example (Fig. S4.2) *CaMKI/calmodulin* may be a mix of both UTR and lncRNA, yet the element is poorly modeled. The remaining four examples have evidence of lncRNA loci detached or reversed from cdsRNA. Example (Fig. S4.5) *dunce/cAMP-specific 3',5'-cyclic phosphodiesterase* has the most complete lncRNA annotation, while examples (Fig S4.1) *ELAV-2 RNA-binding protein*, (Fig S4.4) *odd-skipped* and (Fig S4.6) *exd/homeobox protein extradenticle* have the strongest, longest un-annotated lncRNA expression. See example below.

NCBI Apis annotated lncRNA locus associated with exd, from

https://www.ncbi.nlm.nih.gov/nuccore/NC_007072.3?report=genbank&from=1219147&to=1220526

gene complement(<1..>1380)

/gene="exd"

/note="Derived by automated computational analysis using

gene prediction method: Gnomon."

/db_xref="BEEBASE:GB51904"

/db_xref="GeneID:408763"

gene 1..1380

/gene="LOC102655789"

/note="Derived by automated computational analysis using

gene prediction method: Gnomon."

/db_xref="GeneID:102655789"

ncRNA join(1..93,171..1380)

/ncRNA_class="lncRNA"

/gene="LOC102655789"

/product="uncharacterized LOC102655789"

/note="Derived by automated computational analysis using

gene prediction method: Gnomon. Supporting evidence

includes similarity to: 100% coverage of the annotated

genomic feature by RNA-seq alignments, including 6 samples

with support for all annotated introns"

/transcript_id="XR_407634.1"

/db_xref="GI:571516541"

/db_xref="GeneID:102655789"

**Table S4-1**. Co-located coding UTR and non-coding models of *Drosophila melanogaster* genes from supplemental tables of reference [50] (UTR of named genes) and [51] (lincRNA).

| Gene / locus ID | Chromosome | Location |
| --- | --- | --- |
| lincRNA.S701 | 2L | 9292664-9295537 |
| Ggamma30A | 2L | 9295531 + |
| lincRNA.S1565 | 2L | 19178520-19179570 |
| brat | 2L | 19179758 + |
| Hr39 | 2L | 21259516 + |
| lincRNA.S1749 | 2L | 21259865-21260108 |
| lincRNA.S2634 | 2R | 8162813-8166304 |
| Cam | 2R | 8166314 + |
| lincRNA.427 | 3L | 3714024-3714673 |
| CG32264 | 3L | 3714807 - |
| lincRNA.S4061 | 3L | 3990581-3993983 |
| scrt | 3L | 3994003 + |
| lincRNA.S5070 | 3L | 13504423-13504650 |
| CG17689 | 3L | 13504659 + |
| lincRNA.610 | 3L | 18478111-18479388 |
| AlCR2 | 3L | 18478224 - |
| yata | 3R | 25580172 + |
| lincRNA.895 | 3R | 25580586-25588445 |
| lincRNA.921 | 4 | 475388-479955 |
| CaMKI | 4 | 477426 + |
| lincRNA.922 | 4 | 599121-602267 |
| Ephrin | 4 | 600748 + |
| lincRNA.927 | 4 | 1029685-1033999 |
| plexA | 4 | 1030518 - |
| lincRNA.983 | X | 12578209-12579459 |
| Smr | X | 12578235 - |
| CG15760 | X | 13570265 + |
| lincRNA.989 | X | 13570601-13570882 |
| lincRNA.S9404 | X | 15890250-15890499 |
| exd | X | 15890556 + |

**Table S4-2.** *Nasonia* OGS2 non-coding loci with significant differential expression reported in [54] Tables S8-9. Examination of genome maps for these loci shows no coding sequence evidence, but long (4-10 Kb) spans of strong expression of multiple forms, without intron evidence, that lie intermediate between coding genes (often conserved orthologs), and of similar ambiguous structure as the example locus figures. These may be UTR exons of neighboring genes, or separate transcriptional elements.

**Transcript ID OGS2 ID** comp26431_c0_seq1 Nasvi2EG001246t1 comp15879_c0_seq1 Nasvi2EG001294t1 comp27879_c1_seq1 Nasvi2EG002278t1 comp41510_c0_seq1 Nasvi2EG002279t1 comp37392_c1_seq2 Nasvi2EG002762t1 comp37895_c0_seq1 Nasvi2EG003222t1 comp16006_c0_seq1 Nasvi2EG004018t1 comp41177_c0_seq1 Nasvi2EG004581t1 comp36463_c3_seq1 Nasvi2EG008589t1 comp50390_c0_seq1 Nasvi2EG012180t1 comp90866_c0_seq1 Nasvi2EG001683t1 comp50390_c0_seq1 Nasvi2EG012180t1 comp67785_c0_seq1 Nasvi2EG002805t1 comp424968_c0_seq1 Nasvi2EG008589t1 comp29619_c0_seq1 Nasvi2EG011945t1

**Supplement Figures S4.1 – S4.6 legends**

**Figure S4.1 ELAV-2 RNA-binding protein**

Nasonia Nasvi2EG002573t1; GeneIDs Mouse:15569, Fly:31000, Ant:105423925, Bee:410689; Notes:

Ant: un-annotated 20 KB long3' nc-expression, no introns

Bee: Apis ELAV-2 has un-annotated 20 KB long 3' nc-expression, nointrons

Fly: annotated long 3'utr (8kb vs 4kb cds span), ref-45 has experimental evidence this is UTR rather than other form non-coding expression.

Mouse: complex, hi-express, annotated lncRNA gene at 3'end ofElavl2

**Figure S4.2 CaMKI/Calmodulin**

Nasonia Nasvi2EG007799t1; GeneIDs Mouse:93843, Fly:43792, Ant:105429310, Bee:727249; Notes:

Ant: 4Kb expressed annotated 3'UTR, and internal lncRNAannot

Bee: Apis CaMKI has 1Kb 3'UTR and un-annotated 3Kb long nc expression, no introns

Fly: has 3' detached, un-annotated expression b/n CaMKI and lincRNA CR45126 Mouse: Pnck/CaMKIb2, has un-annotated 3' long nc expression, nointrons

**Figure S4.3 CkIIbeta/casein kinase II beta**

Nasonia Nasvi2EG005276t1; GeneIDs Mouse:13001, Fly:33583, Ant:105424152, Bee:551655; Notes:

Ant: 1Kb annotated 3'utr

Bee: Apis CkIIbeta has annotated 1Kb long 3'UTR, un-annotated 5' 500bp-long nc expression

Fly: has annotated alt-cdsRNA transcripts in 5Kb 3'utr span, has lncRNA CR44962- RB at 5'end,

Mouse: similar to others, Fly model of 3' alt. cdsRNA+UTR is likely

**Figure S4.4 odd-skipped**

Nasonia Nasvi2EG004838t1; GeneIDs Mouse:23967, Fly:32132, Ant:xxxx, Bee: 725053; Notes:

Ant: 500bp long annotated 3'utr, then 5kb long unannotated, detached nc expression Bee: Apis odd has 1kb long annotated 3'utr, then 3Kb long un-annotated detached nc expression

Fly: 800bp annotated 3'utr, then un-annotated, detached lower expression span Mouse: 1Kb annotated 3'utr, then 20Kb annotated 3'lncRNA, and 5kb annotated 5' lncRNA

odd-skipped is in gene "desert" for all these species, no neighbor genes, no transposons, but for flanking expression, annotated only inmouse.

**Figure S4.5 dunce/cAMP-specific 3',5'-cyclic phosphodiesterase**

Nasonia Nasvi2EG011498t1; GeneIDs Mouse:238871, Fly:31309, Ant:105432172, Bee:411288; Notes:

Ant: 500bp annot 3'utr, then 15Kb long, stronger nc expression, part annot lincRNA LOC105432236

Bee: Apis dunce has several annotated, 3' and embedded lncRNAloci

Fly: annotated lncRNA CR44886/FBgn0266180, overlapping 3' end of dunce Mouse: annotated 3' and internal lncRNA.

Dunce has several annotated lncRNA for all these, at 3' end andinternally.

**Figure S4.6 exd/homeobox protein extradenticle**

Nasonia Nasvi2EG003648t1; GeneIDs Mouse:18514, Fly:32567, Ant:105429808, Bee:408763; Notes:

Ant: annotated 3' lncRNA spans exd coding gene without containing coding exons (ie exd intronic to lncRNA), and un-annotated internal strong expression

Bee: Apis exd has 3'UTR embedded lncRNA LOC102655789/XR_407634.1, and 5kb long 3' unannotated ncexpression

Fly: 1.5kb long annotated 3'utr

Mouse: weak 3' expression, but expert-curated 100Kb long lncRNA in 3'region

## Figure S4.1. ELAV-2 RNA-binding protein

## Figure S4.2. CaMKI / calmodulin

## Figure S4.3. CkIIbeta / casein kinase II beta

## Figure S4.4. odd-skipped

## Figure S4.5. dunce / cAMP-specific 3’,5’-cyclic phosphodiesterase

## Figure S4.6. exd / homeobox protein extradenticle
