# Supplementary material for: OGS2: genome re-annotation of the jewel wasp Nasonia vitripennis
Source: BMC Genomics. 2016 Aug 25;17(1):678. doi: 10.1186/s12864-016-2886-9 (PMC5000498; doi:10.1186/s12864-016-2886-9)
Supplement: Additional file 2: Table S1. — Consensus in the location of the OGS2 gene set on the genome assemblies of sibling species Nasonia longicornis and N. giraulti, including recent, high identity paralogs. Almost all OGS2 genes are located on 2 sibling species draft assemblies [4], using GMAP [36] transcript mapping. Paralog locus consensus patterns are tabulated for inparalogs (sharing orthology to other species) and uniquepar (lacking strong homology to other species). Of the total paralog families, each with several genes, most paralogs are on different scaffolds for all species. The counts of tandem paralogs with different separations are indicated. Table S2. A set of 62 orthology groups found in Nasonia transcript assemblies that are poorly mapped onto the current genome, but should be considered as part of a complete Nasonia gene set. Table S3. A total of 75 orthology groups missing from Nasonia but found in 9 other insect genomes. Table S4. Histone genes present in OGS2.0 annotated with presence or absence of lineage-specific expansions. NA entries were not assigned to orthologous groups at the level of Hymenoptera. (ZIP 33 kb) [file 12864_2016_2886_MOESM2_ESM.zip › 12864_2016_2886_MOESM2_ESM/Table S1.doc]

Table S1. Consensus in the location of the OGS2 gene set on the genome assemblies of sibling species *Nasonia longicornis* and *N. giraulti*, including recent, high identity paralogs. Almost all OGS2 genes are located on 2 sibling species draft assemblies [4], using GMAP [36] transcript mapping. Paralog locus consensus patterns are tabulated for inparalogs (sharing orthology to other species) and uniquepar (lacking strong homology to other species). Of the total paralog families, each with several genes, most paralogs are on different scaffolds for all species. The counts of tandem paralogs with different separations are indicated.

| **All Loci *Nasonia vitripennis*** | ***N. longicornus*** | ***N. giraulti*** |
| --- | --- | --- |
| 24,388 | 23,725 | 23,654 |
| **Paralog locus consensus** | **Inparalogs** | **Uniquepar** |
| Count of paralog families (first locus) | 874 | 441 |
| Paralogs on different scaffold | 1795 | 686 |
| Paralogs >10kb distant on same scaffold | 64 | 15 |
| Paralogs <10kb, same orientation, non overlap | 119 | 70 |
| Paralogs <10kb, reversed orientation | 27 | 23 |
| Gene spans overlap (CDS overlap uncertain) | 19 | 8 |
