# Supplementary material for: OGS2: genome re-annotation of the jewel wasp Nasonia vitripennis
Source: BMC Genomics. 2016 Aug 25;17(1):678. doi: 10.1186/s12864-016-2886-9 (PMC5000498; doi:10.1186/s12864-016-2886-9)
Supplement: Additional file 2: Table S1. — Consensus in the location of the OGS2 gene set on the genome assemblies of sibling species Nasonia longicornis and N. giraulti, including recent, high identity paralogs. Almost all OGS2 genes are located on 2 sibling species draft assemblies [4], using GMAP [36] transcript mapping. Paralog locus consensus patterns are tabulated for inparalogs (sharing orthology to other species) and uniquepar (lacking strong homology to other species). Of the total paralog families, each with several genes, most paralogs are on different scaffolds for all species. The counts of tandem paralogs with different separations are indicated. Table S2. A set of 62 orthology groups found in Nasonia transcript assemblies that are poorly mapped onto the current genome, but should be considered as part of a complete Nasonia gene set. Table S3. A total of 75 orthology groups missing from Nasonia but found in 9 other insect genomes. Table S4. Histone genes present in OGS2.0 annotated with presence or absence of lineage-specific expansions. NA entries were not assigned to orthologous groups at the level of Hymenoptera. (ZIP 33 kb) [file 12864_2016_2886_MOESM2_ESM.zip › 12864_2016_2886_MOESM2_ESM/Table S2.doc]

Table S2. A set of 62 orthology groups found in *Nasonia* transcript assemblies that are poorly mapped onto the current genome, but should be considered as part of a complete *Nasonia* gene set*.*

26S proteasome complex subunit DSS1;

28S ribosomal protein S16;

39S ribosomal protein L54;

5'-AMP-activated protein kinase subunit gamma-1;

5'-nucleotidase domain-containing protein 1;

Actin protein 87C;

anon-73B1, protein;

Apoptosis regulator R1;

Apoptosis regulatory protein Siva;

ATP-dependent RNA Helicase DHX33;

bifunctional aminoacyl-tRNA synthetase;

Brain chitinase and chia;

Conserved oligomeric Golgi complex subunit 8;

cytoskeleton-associated protein 5;

Cytosolic iron-sulfur protein assembly protein Ciao1;

DNA replication complex GINS protein PSF1;

Dolichyl-diphosphooligosaccharide--protein glycosyltransferase subunit;

Elongator complex protein 2;

Eukaryotic translation initiation factor 4E;

exocyst complex component 5;

Exonuclease 3'-5' domain-containing protein;

fumarylacetoacetase;

GATA binding protein;

Glycogen synthase;

Importin-9;

Kinesin protein KIF13B;

Mitochondrial intermembrane space import and assembly protein 40-B;

Mitochondrial import inner membrane translocase subunit Tim8

NIF3 protein 1;

Nucleoside diphosphate-linked moiety X motif 18;

Peptidyl-prolyl cis-trans isomerase E;

phosphatidylserine synthase 1;

phosphoglycolate phosphatase;

Polyadenylate-binding protein 2;

POU domain protein CF1A;

Programmed cell death 6-interacting protein;

pyridoxine-5'-phosphate oxidase;

Pyruvate carboxylase;

Ras-related protein M-Ras;

Ras-related protein Rac1;

recQ-mediated genome instability protein 1;

Ribosomal RNA processing protein 36;

Signal peptidase complex subunit 2;

Signal recognition particle 68 kDa protein;

Solute carrier family 46 member;

Sorting nexin-16;

Sorting nexin-6;

Sperm-associated antigen 7;

Spire, protein;

steroid receptor seven-up;

Thioredoxin domain-containing protein 9;

Transcription factor HNF-4;

Transmembrane protein 188;

transport and Golgi organization protein 11;

tRNA-methyltransferase;

Tyrosine-protein kinase CSK;

Ubiquitin-FolD modifier;

Unc-79 , protein;

Uncharacterized aarF domain-containing protein kinase 1;

Uncharacterized protein KIAA0564;

Unknown;

UPF0558 protein C1orf156 protein;

Zinc finger and SCAN domain-containing protein 2;
