# Supplementary material for: OGS2: genome re-annotation of the jewel wasp Nasonia vitripennis
Source: BMC Genomics. 2016 Aug 25;17(1):678. doi: 10.1186/s12864-016-2886-9 (PMC5000498; doi:10.1186/s12864-016-2886-9)
Supplement: Additional file 2: Table S1. — Consensus in the location of the OGS2 gene set on the genome assemblies of sibling species Nasonia longicornis and N. giraulti, including recent, high identity paralogs. Almost all OGS2 genes are located on 2 sibling species draft assemblies [4], using GMAP [36] transcript mapping. Paralog locus consensus patterns are tabulated for inparalogs (sharing orthology to other species) and uniquepar (lacking strong homology to other species). Of the total paralog families, each with several genes, most paralogs are on different scaffolds for all species. The counts of tandem paralogs with different separations are indicated. Table S2. A set of 62 orthology groups found in Nasonia transcript assemblies that are poorly mapped onto the current genome, but should be considered as part of a complete Nasonia gene set. Table S3. A total of 75 orthology groups missing from Nasonia but found in 9 other insect genomes. Table S4. Histone genes present in OGS2.0 annotated with presence or absence of lineage-specific expansions. NA entries were not assigned to orthologous groups at the level of Hymenoptera. (ZIP 33 kb) [file 12864_2016_2886_MOESM2_ESM.zip › 12864_2016_2886_MOESM2_ESM/Table S3.doc]

Table S3. A total of 75 orthology groups missing from *Nasonia* but found in 9 other insect genomes*.*

| **ARP9_Group** | **Group Name** |
| --- | --- |
| ARP9_G13 | RING canal kelch protein; src=aphid_ACYPI000788-PA |
| ARP9_G652 | Dehydrogenase/reductase SDR family member 4; src=apis2ref_XP_395765.2 |
| ARP9_G653 | Unknown; src=bombusimp_XP_003485134.1 |
| ARP9_G873 | Adenylate cyclase type 5; src=bombusimp_XP_003486375.1 |
| ARP9_G907 | sickie, protein; src=bombusterr_XP_003394380.1 |
| ARP9_G1394 | Histone deacetylase complex subunit SAP30; src=bombusterr_XP_003402581.1 |
| ARP9_G1410 | Sodium-dependent dopamine transporter; src=bombusterr_XP_003397466.1 |
| ARP9_G1626 | Unknown; src=apis2ref_XP_624502.3 |
| ARP9_G1627 | Unknown; src=apis2ref_XP_001120879.1 |
| ARP9_G1798 | Lysosomal Pro-X carboxypeptidase; src=bombusimp_XP_003490799.1 |
| ARP9_G1841 | Nuclear hormone receptor HR38, ; src=apis2ref_XP_623628.3 |
| ARP9_G1908 | Zinc finger MYND domain-containing protein 11; src=bombusimp_XP_003485717.1 |
| ARP9_G1977 | Unknown; src=bombusimp_XP_003491598.1 |
| ARP9_G2052 | Potassium voltage-gated channel subfamily H member 7; src=bombusimp_XP_003485015.1 |
| ARP9_G2183 | Very Low-density lipoprotein receptor; src=bombusterr_XP_003395415.1 |
| ARP9_G2262 | mothers against decapentaplegic 6; src=bombusimp_XP_003492241.1 |
| ARP9_G2297 | Tubulin polyglutamylase TTLL4; src=bombusterr_XP_003400542.1 |
| ARP9_G2386 | FAM177A1, protein; src=bombusimp_XP_003490992.1 |
| ARP9_G2595 | Tetraspanin-5; src=bombusterr_XP_003398605.1 |
| ARP9_G2761 | cytosol aminopeptidase; src=bombusterr_XP_003401321.1 |
| ARP9_G2789 | UBX domain-containing protein 1; src=bombusterr_XP_003394852.1 |
| ARP9_G2805 | Transcription factor 21; src=apis2ref_XP_003249110.1 |
| ARP9_G2876 | Serine/threonine-protein kinase GRP; src=bombusimp_XP_003486943.1 |
| ARP9_G2964 | centaurin-gamma-1A; src=bombusterr_XP_003394960.1 |
| ARP9_G3327 | furin Protease 1; src=apis2ref_XP_001121308.2 |
| ARP9_G3528 | charybde, protein; src=bombusterr_XP_003394417.1 |
| ARP9_G3536 | Unknown; src=bombusterr_XP_003397245.1 |
| ARP9_G3778 | RWD domain-containing protein 4; src=apis2ref_XP_394440.3 |
| ARP9_G3998 | tRNA pseudouridine synthase Pus10, ; src=bombusterr_XP_003399044.1 |
| ARP9_G4155 | Tyrosine phosphatase 99A, protein; src=drosmel_NP_651691.4 |
| ARP9_G4182 | F-box/LRR-repeat protein 7; src=apis2ref_XP_001122429.2 |
| ARP9_G4249 | Breast cancer anti-estrogen resistance protein 1; src=bombusimp_XP_003492988.1 |
| ARP9_G4340 | Mitogen-activated protein kinase-binding protein; src=aphid_acyp2eg0002835t1 |
| ARP9_G4787 | Vesicle transport through interaction with T-SNAREs 1A; src=apis2ref_XP_623763.3 |
| ARP9_G5085 | lap1, protein; src=bombusimp_XP_003494347.1 |
| ARP9_G5275 | Usher Syndrome type-1G protein; src=bombusterr_XP_003400917.1 |
| ARP9_G5472 | Myosin heavy chain 95F; src=bombusterr_XP_003399789.1 |
| ARP9_G5531 | High density lipoprotien binding protein / vigilin; src=aphid_ACYPI008252-PA |
| ARP9_G5538 | Unknown; src=bombusimp_XP_003488073.1 |
| ARP9_G5561 | Mitotic spindle assembly checkpoint protein MAD2B; src=anth_E2C532_9HYME |
| ARP9_G5576 | deoxynucleoside kinase; src=bombusterr_XP_003395377.1 |
| ARP9_G5617 | Multidrug resistance-associated protein 7; src=bombusimp_XP_003488656.1 |
| ARP9_G5626 | fem-1 CG6966, protein; src=apis2ref_XP_392810.3 |
| ARP9_G5645 | 72 kDa inositol polyphosphate 5-phosphatase; src=apis2ref_XP_392662.3 |
| ARP9_G5867 | ATP-binding cassette sub-family B member; src=bombusimp_XP_003494648.1 |
| ARP9_G5877 | Ubiquinone biosynthesis protein COQ9; src=apis2ref_XP_624508.1 |
| ARP9_G5883 | phosphatase PTC7 , protein; src=bombusterr_XP_003401636.1 |
| ARP9_G5884 | Cytoplasmic phosphatidylinositol transfer protein 1; src=bombusterr_XP_003403112.1 |
| ARP9_G5915 | CWF19 protein 2; src=bombusterr_XP_003402628.1 |
| ARP9_G5922 | Cation transport regulator protein 1; src=bombusimp_XP_003488209.1 |
| ARP9_G5958 | pancreas transcription factor 1 subunit alpha; src=bombusterr_XP_003403211.1 |
| ARP9_G5966 | Elongation of very long chain fatty acids protein 6; src=bombusimp_XP_003490277.1 |
| ARP9_G5973 | Tyrosine-protein phosphatase non-receptor type 23; src=bombusimp_XP_003487817.1 |
| ARP9_G6059 | Homeobox protein bagpipe; src=anth_E2B3M5_9HYME |
| ARP9_G6116 | E3 ubiquitin-protein ligase MYLIP; src=bombusimp_XP_003485607.1 |
| ARP9_G6120 | Unknown; src=bombusimp_XP_003490683.1 |
| ARP9_G6154 | Dual specificity protein phosphatase 10; src=bombusterr_XP_003395392.1 |
| ARP9_G6158 | Tetratricopeptide repeat protein 25; src=apis2ref_XP_623867.2 |
| ARP9_G6182 | MMS19 nucleotide excision repair protein; src=bombusimp_XP_003490594.1 |
| ARP9_G6191 | Ankyrin repeat domain-containing protein 6; src=apis2ref_XP_391938.4 |
| ARP9_G6282 | 2',5'-phosphodiesterase 12; src=bombusimp_XP_003488973.1 |
| ARP9_G6296 | ADP-ribosylation factor protein 6-interacting protein 1; src=bombusterr_XP_003394073.1 |
| ARP9_G6335 | zer-1 , protein; src=apis2ref_XP_001121426.2 |
| ARP9_G6355 | CAS1 domain-containing protein 1; src=apis2ref_XP_395026.2 |
| ARP9_G6563 | EF-hand domain-containing family member A2; src=bombusterr_XP_003399648.1 |
| ARP9_G6569 | BAI1-associated protein 3; src=apis2ref_XP_392442.4 |
| ARP9_G6571 | Acyl-CoA-binding domain-containing protein 5; src=bombusimp_XP_003485199.1 |
| ARP9_G6624 | Growth arrest-specific protein 8; src=bombusterr_XP_003401190.1 |
| ARP9_G6661 | Ubiquitin-conjugating enzyme E2 W, ; src=apis2ref_XP_392626.3 |
| ARP9_G6670 | Unknown; src=apis2ref_XP_396953.2 |
| ARP9_G6870 | Adenosine monophosphate-protein transferase FICD; src=bombusterr_XP_003395623.1 |
| ARP9_G6891 | exportin-6; src=bombusterr_XP_003397643.1 |
| ARP9_G6894 | Zinc finger protein 330; src=bombusimp_XP_003490380.1 |
| ARP9_G6917 | Unknown; src=bombusterr_XP_003393150.1 |
