# Supplementary material for: OGS2: genome re-annotation of the jewel wasp Nasonia vitripennis
Source: BMC Genomics. 2016 Aug 25;17(1):678. doi: 10.1186/s12864-016-2886-9 (PMC5000498; doi:10.1186/s12864-016-2886-9)
Supplement: Additional file 2: Table S1. — Consensus in the location of the OGS2 gene set on the genome assemblies of sibling species Nasonia longicornis and N. giraulti, including recent, high identity paralogs. Almost all OGS2 genes are located on 2 sibling species draft assemblies [4], using GMAP [36] transcript mapping. Paralog locus consensus patterns are tabulated for inparalogs (sharing orthology to other species) and uniquepar (lacking strong homology to other species). Of the total paralog families, each with several genes, most paralogs are on different scaffolds for all species. The counts of tandem paralogs with different separations are indicated. Table S2. A set of 62 orthology groups found in Nasonia transcript assemblies that are poorly mapped onto the current genome, but should be considered as part of a complete Nasonia gene set. Table S3. A total of 75 orthology groups missing from Nasonia but found in 9 other insect genomes. Table S4. Histone genes present in OGS2.0 annotated with presence or absence of lineage-specific expansions. NA entries were not assigned to orthologous groups at the level of Hymenoptera. (ZIP 33 kb) [file 12864_2016_2886_MOESM2_ESM.zip › 12864_2016_2886_MOESM2_ESM/Table S4.doc]

Table S4. Histone genes present in OGS2.0 annotated with presence or absence of lineage-specific expansions. NA entries were not assigned to orthologous groups at the level of Hymenoptera.

| Name | ODB6 OG ID | Expanded? |
| --- | --- | --- |
| histone deacetylase 3 (92%a) | EOG6N8PM3 | No |
| histone-lysine N-methyltransferase SETDB1 (65%a) | EOG6BRV1R | No |
| histone acetyltransferase Tip60 (83%a) | EOG6H44K2 | No |
| jmjC domain-containing Histone demethylation protein 3B (66%A) | EOG669P90 | No |
| Histone-lysine N-methyltransferase NSD2 (45%A) | EOG60GB5Q | No |
| Histone acetyltransferase MYST4 (66%U) | EOG63N5W3 | No |
| Histone-lysine N-methyltransferase. H3 lysine-79 specific (60%U) | EOG612JM9 | No |
| nucleosomal Histone kinase 1 (49%A) | EOG6H18B6 | No |
| jmjC domain-containing Histone demethylation protein (63%A) | EOG69CNPT | No |
| Histone deacetylase complex subunit SAP130 (49%U) | EOG644J2T | No |
| Histone-lysine N-methyltransferase. H3 lysine-9 specific 5 (Fragment) (55%U) | EOG6WSTRM | No |
| Histone-lysine N-methyltransferase PR-set7 (63%A) | EOG6H9W32 | Yes |
| Histone-lysine N-methyltransferase PR-set7 (56%A) | EOG6H9W32 | Yes |
| Non-histone protein 10 (79%U) | EOG65X6CB | No |
| Histone-lysine N-methyltransferase NSD2 (45%A) | NA | NA |
| Histone deacetylase 4 (79%U) | EOG64QRGN | No |
| jmjC domain-containing histone demethylation protein 1. putative (69%a) | EOG6KSN0T | No |
| histone chaperone asf1 (88%a) | EOG64TMRB | No |
| Histone RNA hairpin-binding protein (57%A) | EOG625491 | No |
| histone acetyltransferase type B catalytic subunit. putative (70%a) | EOG69ZW4X | No |
| Histone-lysine N-methyltransferase. H4 lysine-20 specific. putative (19%U) | NA | NA |
| set1/Ash2 histone methyltransferase complex subunit ASH2 (77%a) | EOG66DJHV | No |
| JmjC domain-containing histone demethylation protein 1D (60%U) | EOG6NK99J | No |
| Histone demethylase UTX (92%U) | EOG6D51FJ | Yes |
| Histone demethylase UTX (94%U) | EOG6D51FJ | Yes |
| Histone-lysine N-methyltransferase Suv4-20 (56%A) | EOG64MW6W | No |
| histone acetyltransferase MYST1. putative (80%a) | EOG65QFV4 | No |
| Histone-lysine N-methyltransferase SETMAR (46%A) | EOG61894T | No |
| Histone demethylase JARID1A (73%U) | EOG6X69Q3 | No |
| jmjC domain-containing Histone demethylation protein 2B (50%A) | EOG62JM67 | No |
| jmjC domain-containing Histone demethylation protein 2C (81%A) | EOG64J10T | No |
| Histone-lysine N-methyltransferase SUV39H2 (46%A) | EOG663XT6 | No |
| histone-binding protein Caf1. putative (99%a) | EOG6RBP0X | No |
| histone deacetylase Rpd3 (87%a) | EOG6N2Z47 | No |
| histone deacetylase complex subunit SAP18. putative (81%a) | EOG6BZKK4 | No |
| lysine-specific Histone demethylase 1A (77%A) | EOG61C5C8 | No |
| lysine-specific histone demethylase 1A (83%a) | EOG6905R9 | No |
| Histone-lysine N-methyltransferase pr-set7 (Fragment) (23%U) | NA | NA |
| Histone deacetylase (51%A) | EOG6DJHBF | No |
| Histone-lysine N-methyltransferase Suv4-20 (52%A) | NA | NA |
| histone-arginine methyltransferase CARMER. putative (89%a) | EOG68SF85 | Yes |
| histone-lysine N-methyltransferase E(z) (84%a) | EOG6BZKHM | No |
| histone-arginine methyltransferase CARMER. putative (87%a) | EOG68SF85 | Yes |
| Histone-lysine N-methyltransferase Suv4-20 (52%A) | NA | NA |
| sin3 histone deacetylase corepressor complex component SDS3. putative (84%a) | EOG6VX0NM | No |
| Histone-lysine N-methyltransferase SETD1B (Fragment) (57%U) | EOG6F7M0V | Yes |
